# Human mobility prediction from region functions with taxi trajectories

**DOI:** 10.1371/journal.pone.0188735

**Published:** 2017-11-30

**Authors:** Minjie Wang, Su Yang, Yi Sun, Jun Gao

**Affiliations:** Shanghai Key Laboratory of Intelligent Information Processing, College of Computer Science, Fudan University, Shanghai, China; Beihang University, CHINA

## Abstract

People in cities nowadays suffer from increasingly severe traffic jams due to less awareness of how collective human mobility is affected by urban planning. Besides, understanding how region functions shape human mobility is critical for business planning but remains unsolved so far. This study aims to discover the association between region functions and resulting human mobility. We establish a linear regression model to predict the traffic flows of Beijing based on the input referred to as bag of POIs. By solving the predictor in the sense of sparse representation, we find that the average prediction precision is over 74% and each type of POI contributes differently in the predictor, which accounts for what factors and how such region functions attract people visiting. Based on these findings, predictive human mobility could be taken into account when planning new regions and region functions.

## Introduction

People in cities suffer from increasingly severe traffic jams. Tremendous efforts have been made to study the mechanism regarding how traffic jams are generated. One dominant point is that traffic flows can be regarded as mutually affected components in a complex network; as a result, traffic jams spread out to affect traffic flows in the whole city through local interactions [1]. The endeavor being devoted to human mobility pattern mining and traffic flow prediction has resulted in the following analytical methodologies: Statistical Methods [2][3], Non-negative Matrix Factorization and Optimization Methods [4], Entropy-maximizing Methods [5], Multiscale Radial Basis Function (MSRBF) networks [6], and Deep Learning Methods [7]. However, such studies are limited in that they do not take into account the social contexts in which traffic flows are generated, say, the motivations that drive people to travel from one place to another place. In fact, there are regular patterns to people′s travel behavior and many motivations that may cause people to visit a place at a particular time. For example, most people travel to offices at morning rush hour and go shopping in their leisure time. It is experimentally observed that the daily travel of an individual has a high possibility of falling within deterministic paths [8]. Therefore, awareness of the causes of collective human mobility is the key to alleviating traffic jams in the urban planning stage, which is preferable to regulations that attempt to fix deterministic traffic flows. In addition, the number of visitors to a certain region is of interest to the people to start new business if predictable. This study aims to answer two questions that have never been explored: (1) Do certain region functions lead to predictable human mobility, and is it possible to predict such human mobility from region functions? (2) Is there any deterministic relationship between each type of region function and the attraction of people? If so, how does one quantitatively evaluate the impact factor of every causal of human mobility (e.g., business, residence, entertainment, and shopping)? In this study, we format the problem as sparse representation rendered prediction and optimize the predictor in a manner similar to variable selection. We conduct the experiments with the GPS traces of 12000 taxies in Beijing city for one month. The results are encouraging in that over 74% prediction accuracy has been obtained when taking the POI (point of interest) feature of 42×45 blocks as input. Moreover, the dominant factors contributing to collective human mobility at specified times are identified and quantified, which coincide with the prior knowledge.

## Related work

In early studies, mobile phone data are used for inferring social networks [9] and land survey [10], which advance the studies on social and natural phenomena through human mobility. Recently, region function discovery and land use identification from human mobility have attracted much attention. In [11], blocks in Beijing are grouped into a couple of regions with explicit functions consistent with human knowledge, where human mobility features extracted from taxi trajectories are combined with POI features to infer the attributes of a given block in the framework of Topic Model. Then, by clustering the blocks in accordance with the similarity between the attributes over latent topics, a couple of regions dominated by a certain function are obtained, for instance, diplomatic and embassy, education and science, and commercial/entertainment areas. In [12], the land uses of Manhattan are categorized into 4 classes, say, commercial, residential, industry, and recreation areas, by clustering tweet activities with geo-tags in each land segment, where Self-Organizing Map (SOM) is applied to obtain the segmentation of the land and *k*-means clustering is employed to group the land segments with similar tweet activities. In [3], taxi traces in Shanghai are decomposed into the linear combination of 3 basic patterns by means of Non-negative Matrix Factorization, where the 3 basic patterns correspond with commuting between home and workplace, business traveling between two workplaces, and trips from or to other places. As a result, the land use of the origins and destinations of the traffic flows can be inferred from such basic mobility patterns. In [13], the categories of POI are inferred from the taxi stops in Milan based on Hidden Markov Model. In [14][15][16], supervised methods are applied to classify regions into a couple of categories of different land uses with manually annotated labels a priori for training.

Unlike the aforementioned works, which are focused on region function annotation, our goal is to infer whether there are consistent mappings to correlate people′s collective mobility to the Bags of POI features and to determine how to predict the traffic flows to be generated by newly planned regions through the POI features. Awareness of such issues makes optimization of traffic loads at the urban planning stage possible. In [17], the correlation between human mobility patterns and region functions is mined through co-training based multi-view clustering, but the association between the two modalities of the data holds in about 50% of cases only. However, the aforementioned study does not solve the following two problems: (1) How to discover the impact factor of each region function in terms of affecting human mobility. (2) How to predict the corresponding traffic flows from the functions of a region. This study proposes to solve the two problems in the framework of sparse representation. In the author′s previous work [18], all regions of the city of interest are divided into hot regions and non-hot regions in a coarse manner according to the population flow in different time periods. In this study, fine-grained region partitions are considered in contrast to [18]. Moreover, the impact of the city functions of neighboring regions in terms of their effect on the human mobility of the target region is investigated in detail. The varying parameters applied to region partition and analysis of the influence of neighboring regions lead to more extensive experiments over [18].

The rest of the paper is organized as follows: In section 3, we propose details about predicting human mobility from region POIs. The experimental findings under different cases are presented in section 4. Discussion and conclusive remarks are provided in section 5.

## Predicting human mobility from POI

**Fig 1:**
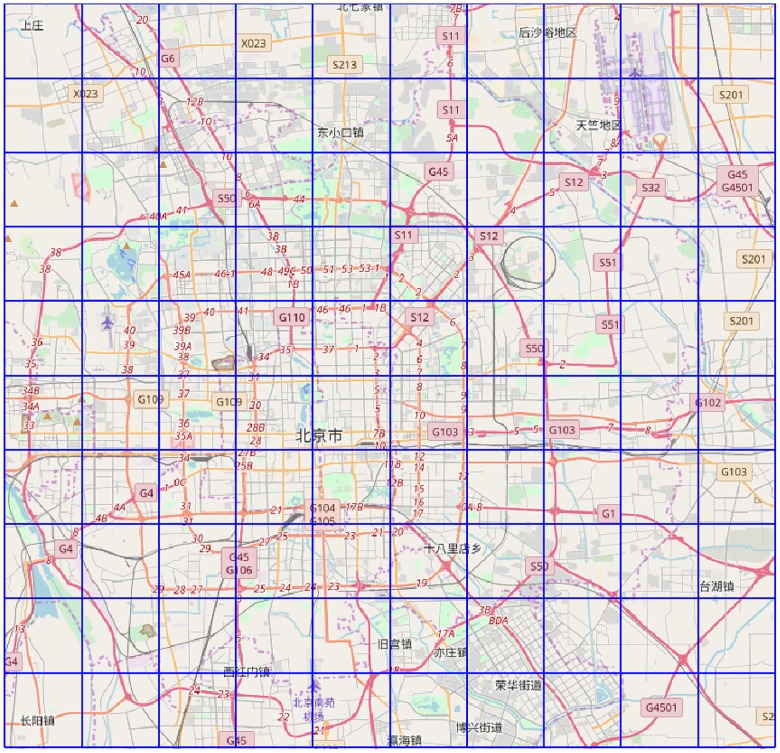
Partition of Beijing into 1km×1km blocks.

With the continuous development of urban construction, all kinds of living, working, and entertainment facilities have been continuously added to a city′s different regions to configure various functions for every region. So far, the city functions enabled by such facilities can mainly be sorted into 20 categories: life services, corporations/enterprises, government agencies and social organizations, shopping services, scenic spots, and so on, as shown in Table 1. The various types of city functions with geo-tags are commonly known as POIs (points of interest), where each original POI record includes the name and the category of the place, and the latitude and the longitude of the position. In order to study the differences between regions in a city, a grid is applied to divide the urban area into a couple of regions. Taking Beijing City as an example, as illustrated in Fig 1 with OpenStreetMap, the city is partitioned into a couple of regions, and the functions of each region are in fact an assembly of the POIs listed in Table 1.

**Table 1:** POI categories.

No.	POI Category	No.	POI Category
1	Fitness Center	11	Car Service
2	Life Service	12	Scenic Spot
3	Corporation & Business	13	Transportation Facility
4	Government & organization	14	Residence
5	Restaurant	15	Car Maintaining
6	Hospital	16	Well-known Address
7	Shopping Mall	17	Car Sales
8	Financial Service	18	Motor Service
9	Hotel	19	Public Service
10	Education & Training	20	Road/Street & Entrance

### Bag-of-Words representation of city functions

For every region as shown in Fig 1, we collect the POI records through the free software, namely, Baidu API. Here, what we are concerned with is the city function denoted by every POI record, and as shown in Table 1, there are 20 POI categories in total. Hereafter, we characterize the city functions of every region through the Bag of Words model, which is well-known in the literature of Natural Language Processing. That is, for region *r*, the corresponding Bag of POI (BOP) representation is a vector in the form of

$$F_{r}=\left(p_{1}^{r},p_{2}^{r},\ldots ,p_{n}^{r}\right)$$

where $p_{i}^{r}$ represents the number of points belonging to the *i*th POI category in Table 1 and *n*=20 the total 20 POI categories.

### Statistics of origin-destination flows

Origin-destination (OD) flows, which count the number of individual movements between locations in a city, reflect not only human activity but also urban dynamics, and they are widely used in city planning and traffic engineering [19]. In this paper, we estimate the regional OD flows during different time periods from all taxi traces in the city of interest. Here, $O_{t}^{r}$ and $D_{t}^{r}$ are used to represent the outflow and inflow of region *r* during time period *t*.

### Identifying hot regions

From the perspective of city planning or transportation management, the regions with large outflows and inflows should receive much more attention. Here, such regions are referred to as hot regions. We apply 2 thresholds to the inflow and the outflow of every region to select the hot regions of a given time duration, which are defined in Eqs (1) and (2) as follows:

$$\begin{array}{c} H_{r,D,t}=\{\begin{array}{cc} 1 & D_{t}^{r}>thr_{D,t}\\ 0 & else \end{array} \end{array}$$ (1)

$$\begin{array}{c} H_{r,O,t}=\{\begin{array}{cc} 1 & O_{t}^{r}>thr_{O,t}\\ 0 & else \end{array} \end{array}$$ (2)

where *H*_*r*,*D*,*t*_ and *H*_*r*,*O*,*t*_ indicate whether region *r* is a hot region during time period *t*, and *thr*_*D*,*t*_ and *thr*_*O*,*t*_ are the thresholds to meet the following requirements:

$$\begin{array}{c} \sum_{D_{t}^{r}>thr_{D,t}}D_{t}^{r}>\alpha \sum_{r=1}^{N}D_{t}^{r} \end{array}$$ (3)

$$\begin{array}{c} \sum_{O_{t}^{r}>thr_{O,t}}O_{t}^{r}>\beta \sum_{r=1}^{N}O_{t}^{r} \end{array}$$ (4)

where *α* and *β* are the two parameters to be adjusted empirically and *N* the number of regions in the city of interest. Smaller values of *α* and *β* correspond with a higher degree of hotness of the hot regions.

### Discovering correlation between region functions and human mobility

During time period *t*, we can get the population entering and leaving the hot regions via Eqs (1) and (2), and we formulate the inflows and outflows of all hot regions as follows:

$$Y_{O,t}={\left[O_{t}^{1},O_{t}^{2},\ldots ,O_{t}^{m}\right]}^{T}$$

$$Y_{D,t}={\left[D_{t}^{1},D_{t}^{2},\ldots D_{t}^{k}\right]}^{T}$$

where *m* and *k* are the numbers of the hot regions in terms of outflows and inflows identified via Eqs (3) and (4), respectively. Meanwhile, the corresponding BOP representation of the city functions of such hot regions can be formatted into two matrices as follows:

$$X_{O,t}=[\begin{array}{cccc} p_{1}^{1} & p_{2}^{1} & \ldots & p_{n}^{1}\\ p_{1}^{2} & p_{2}^{2} & \ldots & p_{n}^{2}\\ \ldots & \ldots & \ldots & \ldots \\ p_{1}^{m} & p_{2}^{m} & \ldots & p_{n}^{m} \end{array}]$$

$$X_{D,t}=[\begin{array}{cccc} p_{1}^{1} & p_{2}^{1} & \ldots & p_{n}^{1}\\ p_{1}^{2} & p_{2}^{2} & \ldots & p_{n}^{2}\\ \ldots & \ldots & \ldots & \ldots \\ p_{1}^{k} & p_{2}^{k} & \ldots & p_{n}^{k} \end{array}]$$

We suppose that there is a linear relation to correlate the region functions with outflows/inflows as follows:

$$\begin{array}{c} Y_{O,t}=X_{O,t}\cdot W_{O,t} \end{array}$$ (5)

$$\begin{array}{c} Y_{D,t}=X_{D,t}\cdot W_{D,t} \end{array}$$ (6)

where *W*_*O*,*t*_ and *W*_*D*,*t*_ are two *n*-dimensional vectors composing the weighting coefficients to be solved. Once *W*_*O*,*t*_ and *W*_*D*,*t*_ are obtained, we can then know how much each of the *n* POI categories contributes to generate the corresponding portions of the inflows and outflows in regard to all the hot regions as a whole. Here, sparse representation method (SRM) is used to solve Eqs (5) and (6) since the method leads to as few as possible nonzero weights in *W*_*O*,*t*_ and *W*_*D*,*t*_ given a predefined precision of approximation. In detail, we use Least Angle Regression method (LARS) algorithm [20] to obtain the estimation of *W*_*O*,*t*_ and *W*_*D*,*t*_ as follows:

$${\hat{W}}_{O,t}=\underset{W_{O,t}}{\arg\min}\left\{\lambda_{1}∥W_{O,t}{∥}_{1}+\frac{1}{2}∥Y_{O,t}-X_{O,t}W_{O,t}{∥}_{2}^{2}\right\}$$ (7)

$${\hat{W}}_{D,t}=\underset{W_{D,t}}{\arg\min}\left\{\lambda_{2}∥W_{D,t}{∥}_{1}+\frac{1}{2}∥Y_{D,t}-X_{D,t}W_{D,t}{∥}_{2}^{2}\right\}$$ (8)

where *λ*_1_ and *λ*_2_ are the Lagrange multipliers to control the balance of the penalty to the two terms, say, the sparseness of the solution and the precision of approximation. Due to the constraints of ‖*W*_*O*,*t*_‖ and ‖*W*_*D*,*t*_‖ imposed on the optimization indices defined in Eqs (7) and (8), we can obtain as few as possible nonzero weights in ‖*W*_*O*,*t*_‖ and ‖*W*_*D*,*t*_‖, corresponding with the relevant variables in the POI categories contributive to the prediction. Meanwhile, the numerical values of such nonzero weights reflect how big roles they play in the predictor, that is, how much each relevant POI category contributes to the prediction.

### Preprocessing

Prior to mining the correlation between region functions and human mobility, some preprocessing steps are necessary. Intuitively, except for the motivation to attract people to the destination region, the functions of the neighboring regions should also have an impact on people′s travel behaviors to some extent. Therefore, we absorb the city functions of the neighboring regions into the BOP representation of every region; that is, we compute the *i*th component of the new BOP representation of region *r* as:

$$\begin{array}{c} {\bar{p}}_{i}^{r}=\kappa .p_{i}^{r}+\left(1-\kappa \right)\sum_{r^{\prime }\in R}p_{i}^{r^{\prime }} \end{array}$$ (9)

where *R* is the collection of the eight neighboring regions around region *r*, and *κ* is the factor to weight the contribution of region *r* and the 8 neighboring regions. A bigger *κ* corresponds with a smaller contribution of the neighboring regions. Then, the term frequency-inverse document frequency (TF-IDF) method is applied to the new descriptor after absorbing the city functions of the neighboring regions, namely, ${\bar{F}}_{r}={\left[{\bar{p}}_{1}^{r},{\bar{p}}_{2}^{r},...{\bar{p}}_{n}^{r}\right]}^{T}$. Here, the TF term of the *i*th POI category is defined as:

$$\begin{array}{c} TF_{i}^{r}=\frac{{\bar{p}}_{i}^{r}}{\sum_{i=1}^{n}{\bar{p}}_{i}^{r}} \end{array}$$ (10)

The IDF terms of the *i*th POI category are defined as:

$$\begin{array}{c} IDF_{i}^{r}=\log\frac{\left|r\right|}{|r_{i}|}+\delta \end{array}$$ (11)

where |*r*| is the number of hot regions in the time duration of interest, |*r*_*i*_| the number of hot regions containing the *i*th POI category, and *δ* = 0.000001 a minimum value to avoid $IDF_{i}^{r}=0$. Then, we can compute the TF-IDF value of the *i*th POI category in region *r* as follows:

$$\begin{array}{c} TI_{i}^{r}=TF_{i}^{r}\cdot IDF_{i}^{r} \end{array}$$ (12)

After the above preprocessing, we can get the new region function description for region *r*:

$$F_{r}^{\prime }=\left[TI_{1}^{r},TI_{2}^{r},...,TI_{n}^{r}\right]$$

For the *m* hot regions at time *t*, correspondingly, we get the overall descriptor in the form of a matrix:

$$X_{O,t}^{\prime }=[\begin{array}{cccc} TI_{1}^{1} & TI_{2}^{1} & \ldots & TI_{n}^{1}\\ TI_{1}^{2} & TI_{2}^{2} & \ldots & TI_{n}^{2}\\ \ldots & \ldots & \ldots & \ldots \\ TI_{1}^{m} & TI_{2}^{m} & \ldots & TI_{n}^{m} \end{array}]$$

Similarly, we can obtain $X_{D,t}^{\prime }$. Then, $X_{O,t}^{\prime }$ and $X_{D,t}^{\prime }$ will be applied to Eqs (7) and (8) to obtain the solution.

## Experiments

### Performance evaluation metric

In the training stage, after applying $X_{O,t}^{\prime }$ and $X_{D,t}^{\prime }$ to Eqs (7) and (8), we obtain the weights ${\hat{W}}_{O,t}$ and ${\hat{W}}_{D,t}$. In the testing stage, we apply ${\hat{W}}_{O,t}$, ${\hat{W}}_{D,t}$, $X_{O,t}^{\prime }$ and $X_{D,t}^{\prime }$ to Eqs (5) and (6) to get the predicted outflows and inflows. Here, the Mean Absolute Percentage Error (MAPE) is used to measure the prediction accuracy, that is,

$$\begin{array}{c} Accuracy_{O}=1-\frac{1}{m}\sum_{r=1}^{m}|\frac{O_{t}^{r}-{\hat{O}}_{t}^{r}}{O_{t}^{r}}| \end{array}$$ (13)

$$\begin{array}{c} Accuracy_{D}=1-\frac{1}{k}\sum_{r=1}^{k}|\frac{D_{t}^{r}-{\hat{D}}_{t}^{r}}{D_{t}^{r}}| \end{array}$$ (14)

where ${\hat{O}}_{t}^{r}$ and ${\hat{D}}_{t}^{r}$ are the predicted outflow and inflow for region *r*, *m* and *k* the number of hot regions at time *t* in terms of outflow and inflow, respectively.

### Parameter setting

According to Eqs (7) and (8), the only parameters to be set are *λ*_1_ and *λ*_2_. By trying *λ*_1_,*λ*_2_ ∈ [0.001,0.3] with an increment of 0.002, we can obtain the weights and the prediction accuracy in the sense of Eqs (13) and (14) for every parameter value, where we set the parameter value as that leading to the highest precision on the training data.

### The data

**Fig 2:**
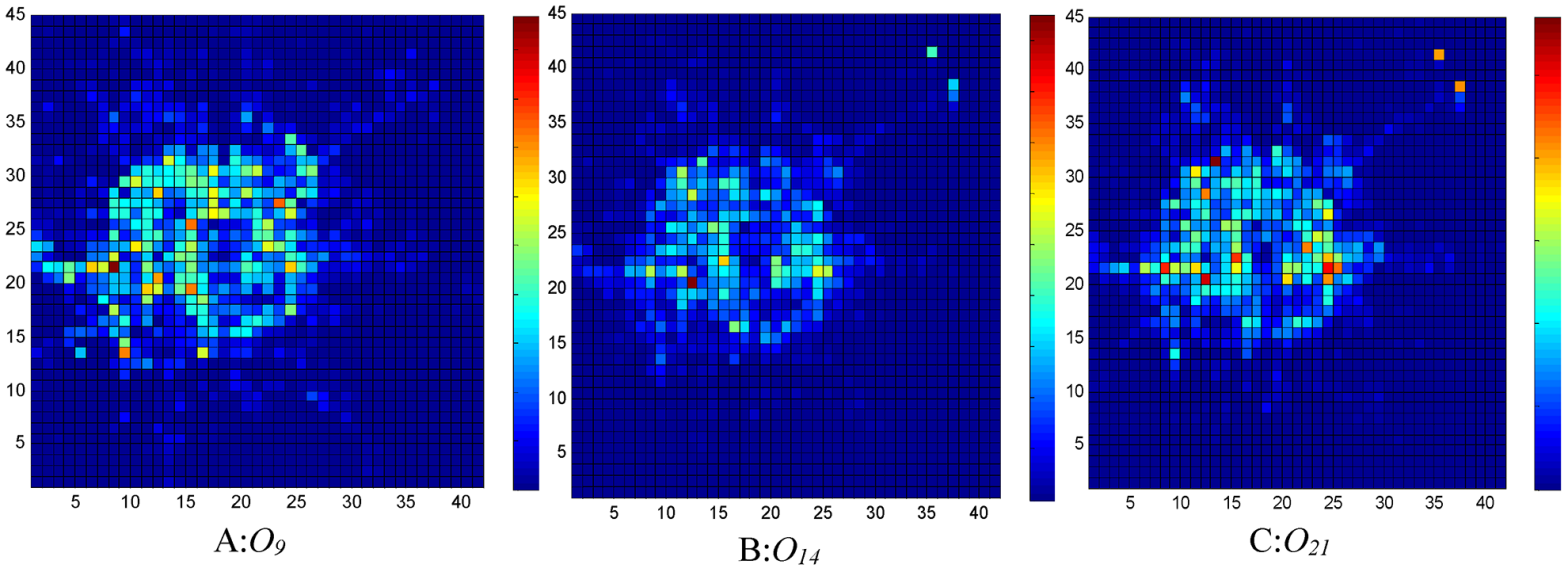
Distribution of outflows at different time intervals. A: 8:00-9:00; B: 13:00-14:00; C: 20:00-21:00.

**Fig 3:**
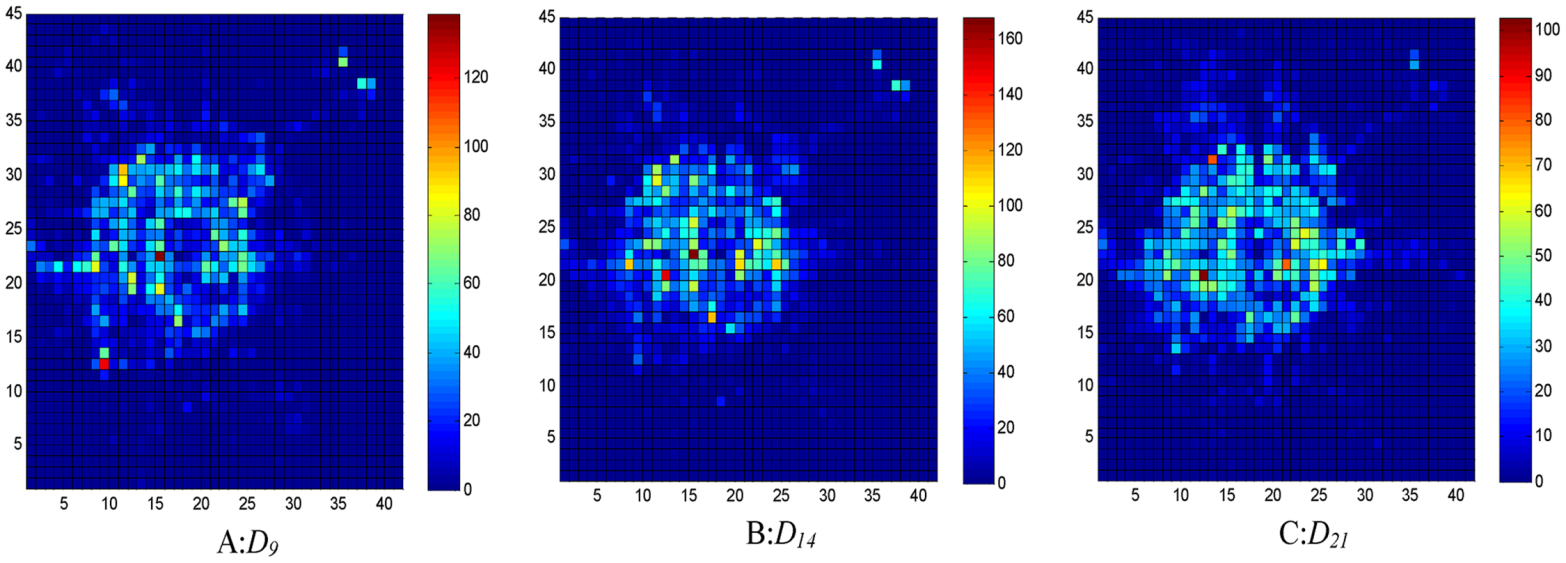
Distribution of inflows at different time intervals. A: 8:00-9:00; B: 13:00-14:00; C: 20:00-21:00.

The data of about 12000 taxies with GPS trajectories traveling within the urban area of Beijing City are used in the experiments. The data are mainly collected from 1 to 31 October 2012, and the data of OD flows are extracted from the origins and destinations of taxi journeys with passengers. We take every hour of each day as a time period to compute the outflows and inflows. The sampling frequency of each GPS trace is 1−2 times per minute. The data contains the following items: TAXI_ID, GPS_TIME, GPS_LONGITUDE, GPS_LATITUDE, and STATE, which indicates whether a taxi has passengers. A filtering process is adopted to remove the incorrect records that are caused by hardware faults. In addition, the records out of the area of interest are also discarded. Distribution of the traffic flows throughout the city at different time intervals, say, morning rush hour, working hours, and evening rush hour, on 15 October 2012 are shown in Figs 2 and 3, where vibrant colors correspond to large traffic flows. As can be seen, the traffic flows at the city center area are dense at morning rush hour since more bright regions are visible. During the afternoon working hours, the traffic flows distribute more uniformly, and there are relatively fewer bright regions. Although the number of bright regions at evening leisure time is less than that of morning rush hour, redder regions indicating high traffic volume are present. Based on Eqs (1)–(4), hot regions at a certain time interval of every day can be quickly identified.

### Findings

In the experiments, as for training, we first select 20 days in a month at random to get the hot regions in the sense of Eqs (1)–(4) for all the time periods. Then, the data of the remaining 11 days are used to validate the predictor. After that, the data for work days and weekends are applied separately for performance evaluation. Finally, the traffic patterns in different week days are investigated.

**Fig 4:**
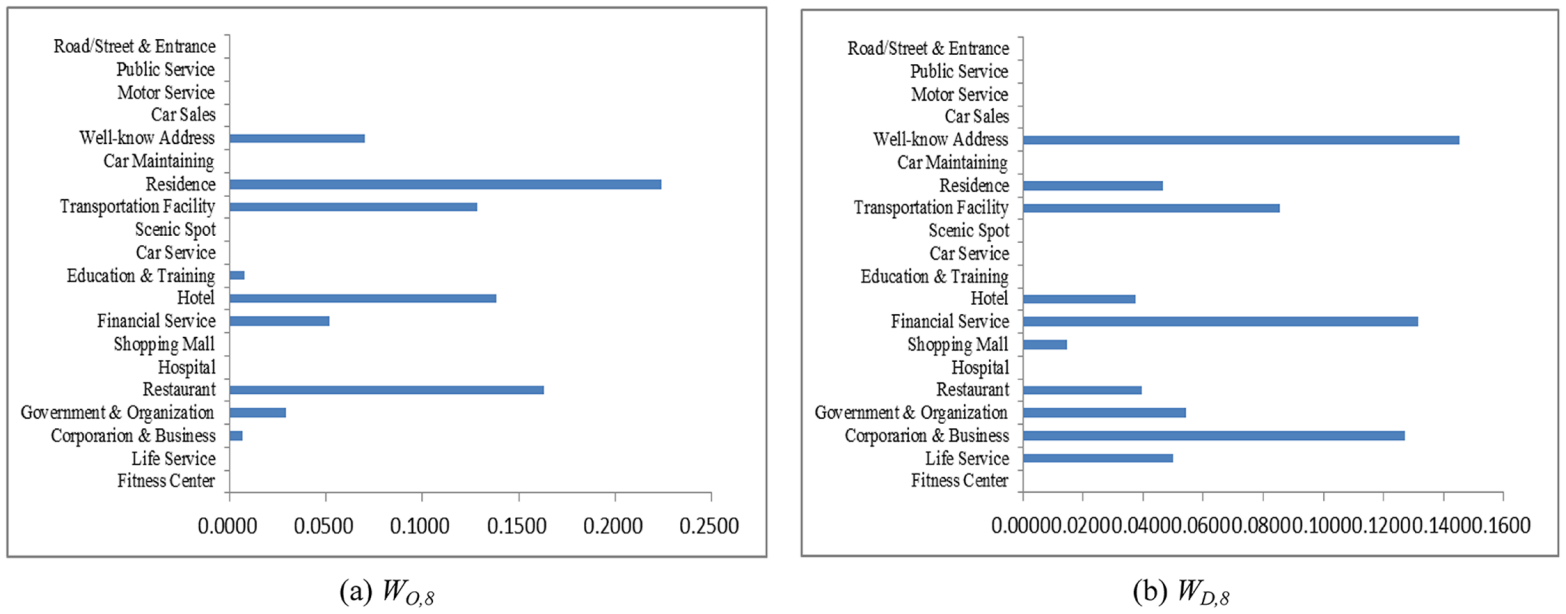
The weights contributing to the traffic flows in 7:00-8:00 AM. (a) Outflow; (b) Inflow.

**Fig 5:**
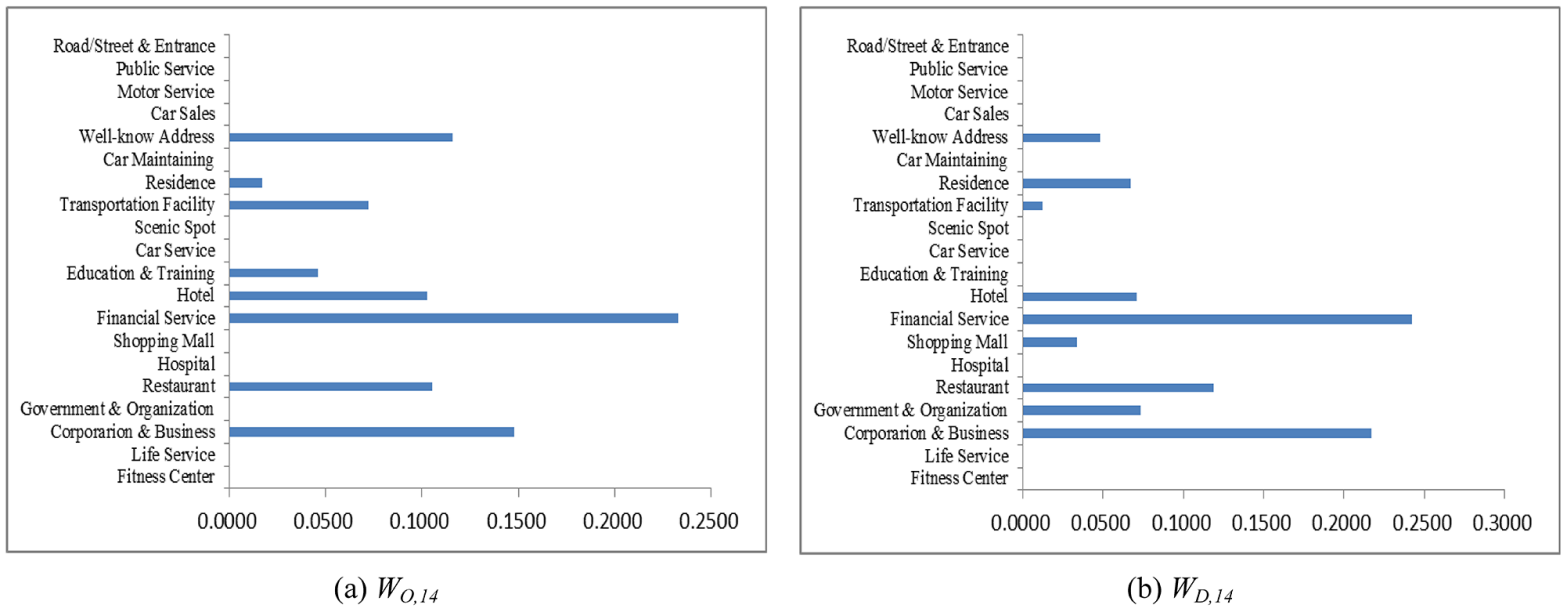
The weights contributing to the traffic flows in 13:00-14:00 PM. (a) Outflow; (b) Inflow.

**Fig 6:**
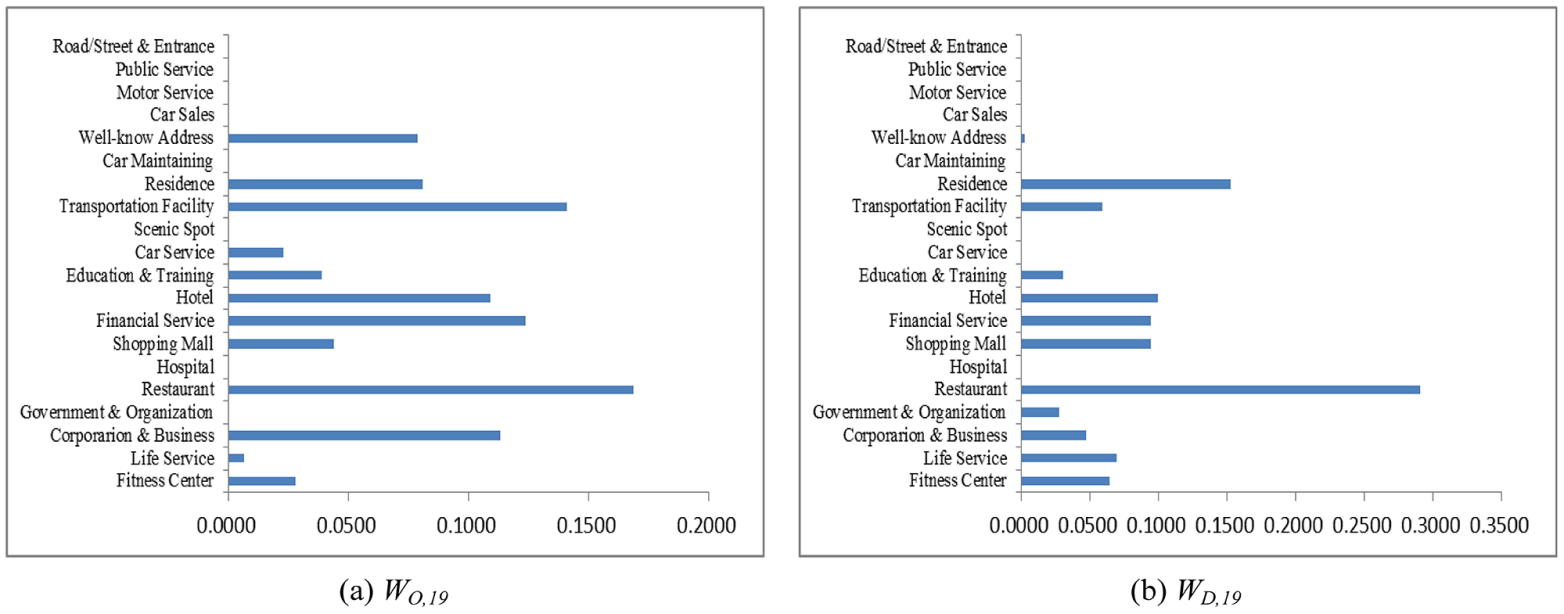
The weights contributing to the traffic flows in 18:00-19:00 PM. (a) Outflow; (b) Inflow.

**Fig 7:**
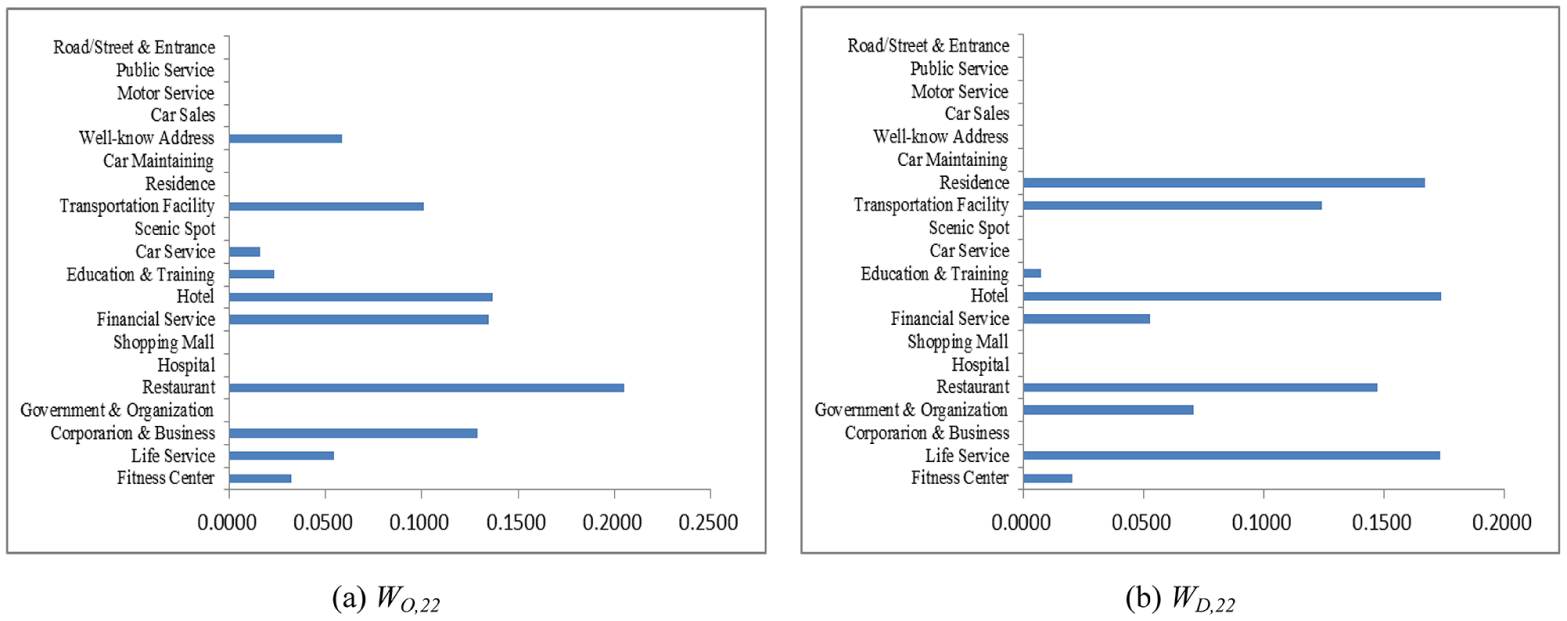
The weights contributing to traffic flows in 21:00-22:00 PM. (a) Outflow; (b) Inflow.

As an example, let the POI weighting factor to absorb neighboring regions be *κ* = 7/8 and the hot region selection parameters be *α* = 1/3 and *β* = 1/3. Counting the taxi OD flows as a snapshot of human mobility, the weighting coefficients solved from Eqs (7) and (8) at different time periods are shown in Figs 4–7. The physical meanings of the dominant weights are explained though the 3 examples as follows: It is known that people usually leave home for office in the morning rush hour, travel between different work places during the day and return home or go out for entertainment in the evening. The human activities reflected in Figs 4–7 are consistent with such prior knowledge. It can be observed in Fig 4 that during the morning rush hour from 7:00 AM to 8:00 AM, outflows are mainly from residential areas, restaurants, and hotels, while the destinations are mostly corporations, financial service agencies, and some well-known addresses, some of which should correspond to transportation hubs. As shown in Figs 5 and 6, for lunch time and dinner time (13:00 PM − 14:00 PM and 18:00 PM − 19:00 PM), notably, the main origin is corporation/business, and the main destination is restaurants. At night from 21:00 PM to 22:00 PM, the main traffic flows are from restaurants to the main destinations of residential areas and hotels, as shown in Fig 7. The aforementioned 3 cases are consistent with the prior knowledge regarding human mobility, which verifies the reasonableness of the solution.

**Fig 8:**
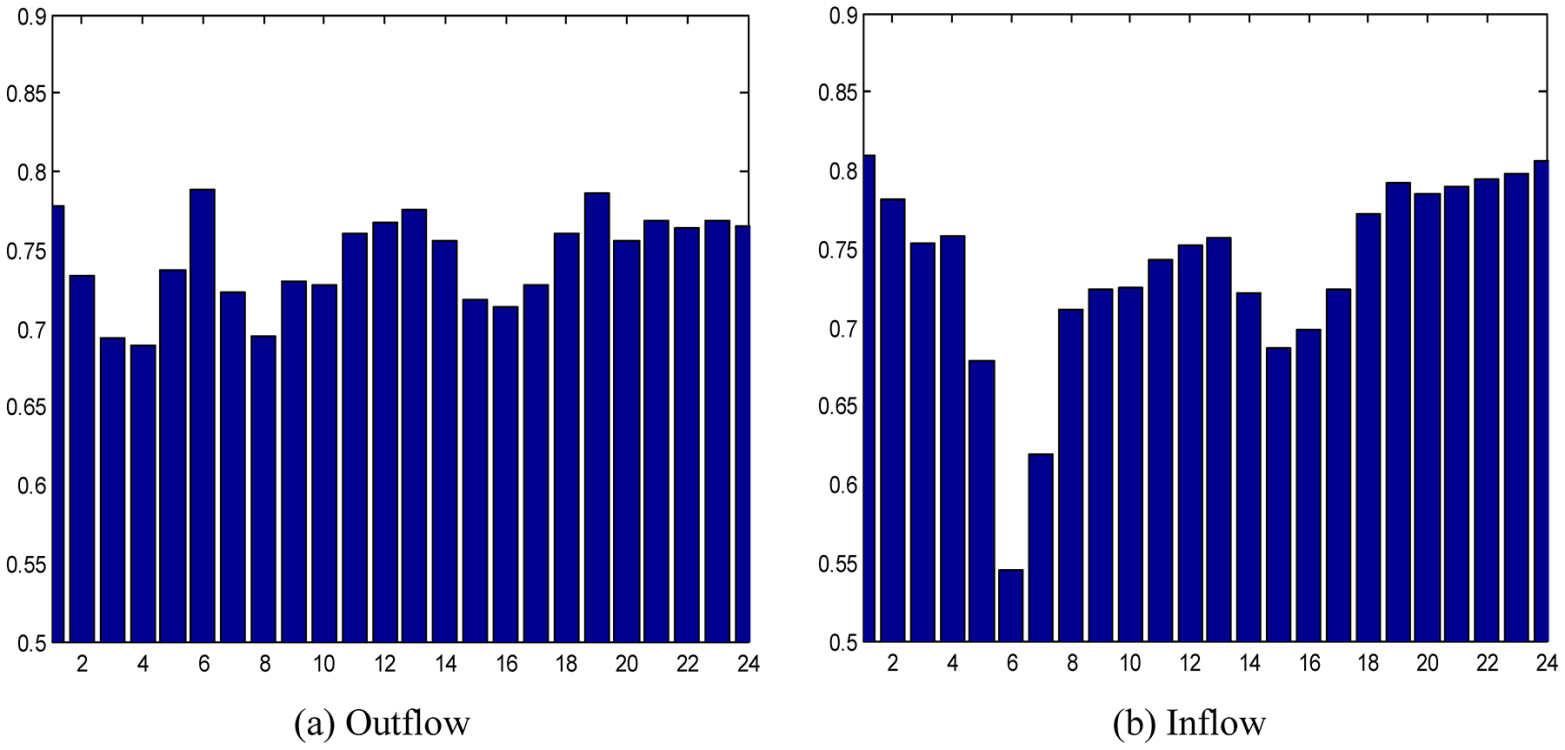
The average prediction accuracies at different time periods. (a) The case of outflow; (b) The case of inflow.

The average prediction accuracies of different time periods are shown in Fig 8, in which most of the prediction accuracies are greater than 0.7 and only a few are between 0.5 and 0.65. Especially at 5:00 AM−6:00 AM, the average prediction accuracy for inflows is only about 54%. After that period, the prediction accuracy is much better. Since 5:00 AM−6:00 AM is not the normal time duration for people to start their daily lives, travel is characterized by greater uncertainty and the OD data are sparse, which causes the lower prediction accuracy. From 8:00 AM, prediction accuracy gradually increases and reaches the highest point at 13:00 PM. During working hours, travel behaviors tend to be random, causing prediction accuracy to degrade. When evening rush hour begins at 16:00 PM, prediction accuracy starts to increase again and reaches another peak at the end of evening rush hour (19:00 PM). Then, high precision is maintained until 24:00. The overall average prediction accuracy for an entire day is 74.50%.

**Fig 9:**
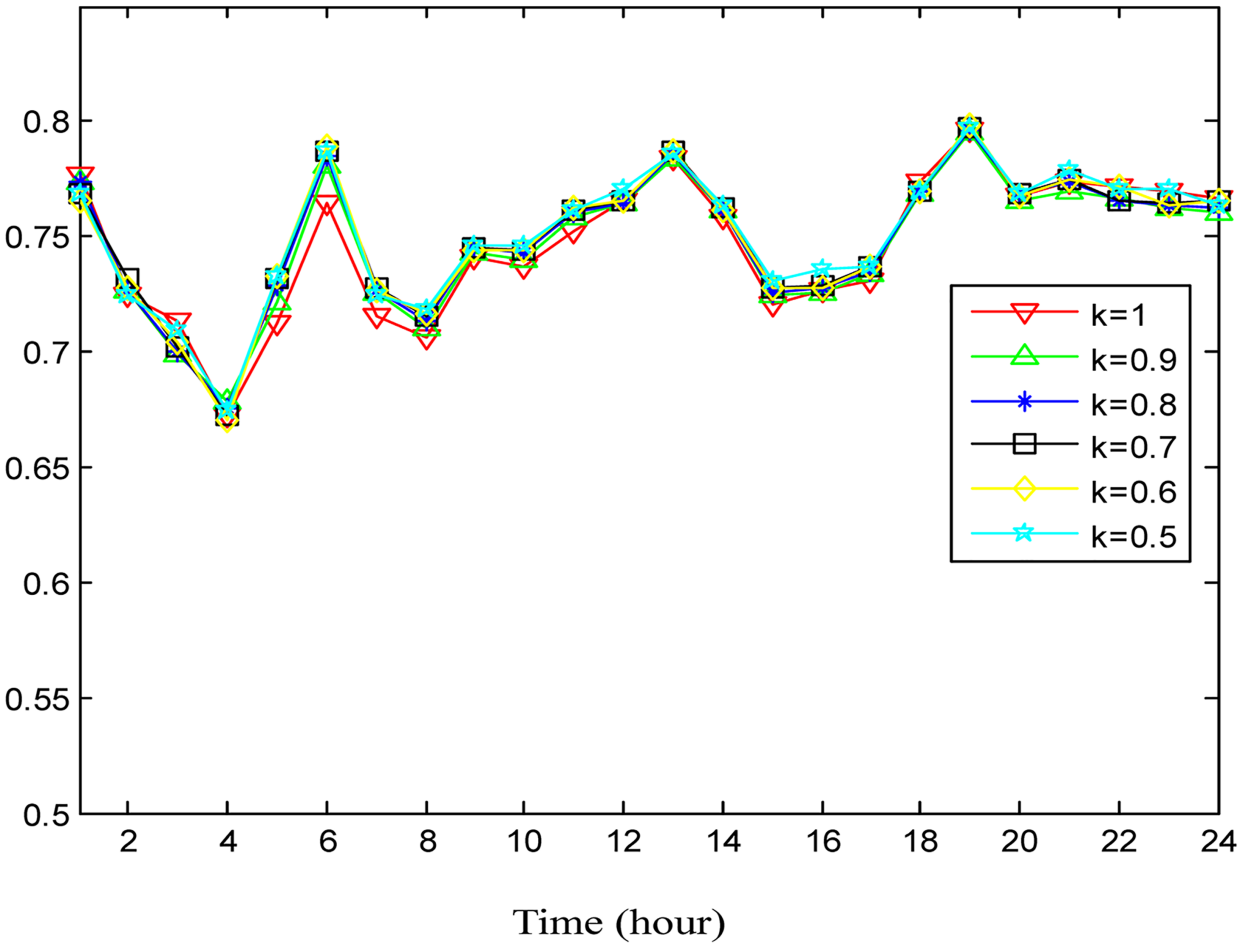
The average prediction accuracies for outflow under different *κ* in different time durations.

**Fig 10:**
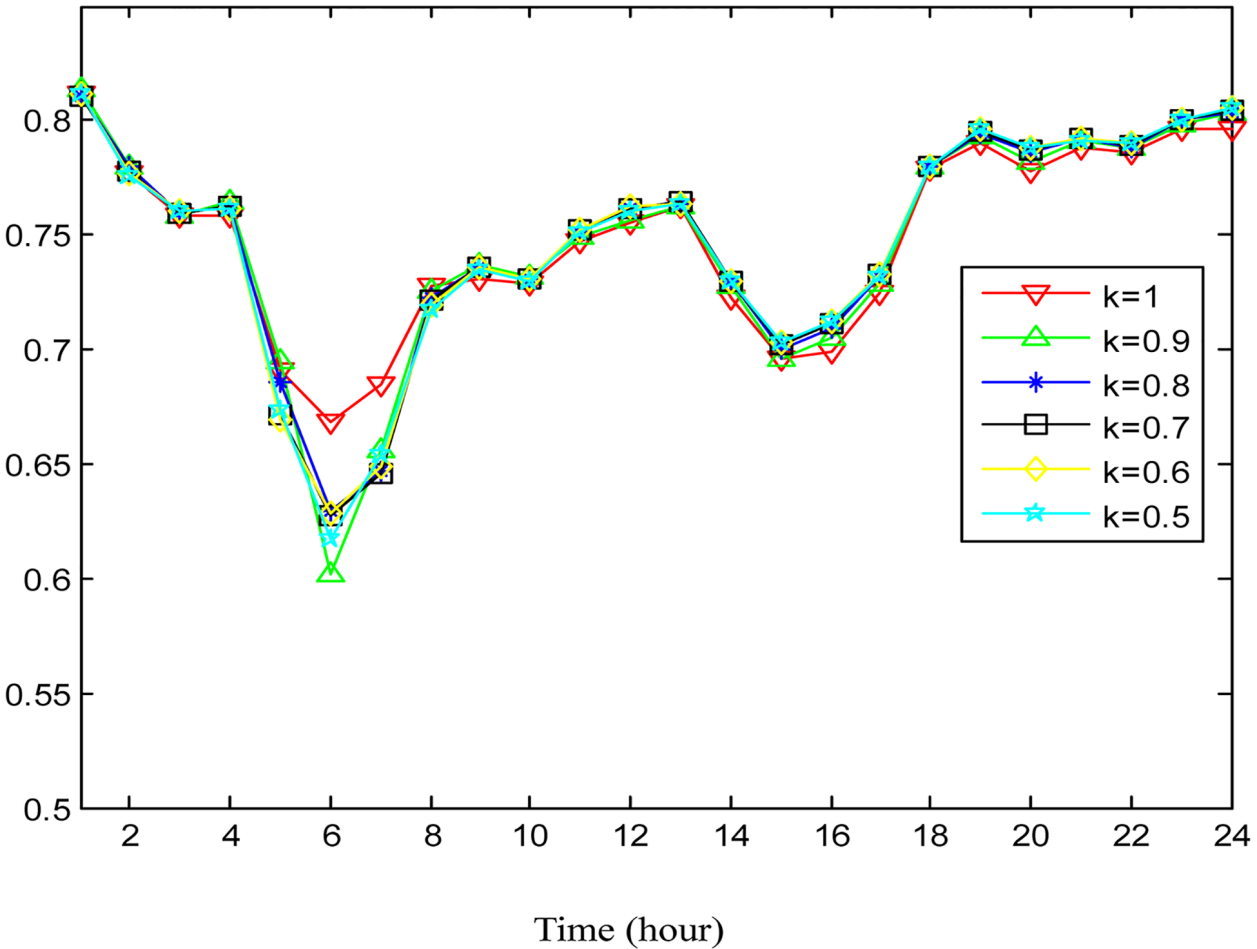
The average prediction accuracies for inflow under different *κ* in different time durations.

In order to study the effect of absorbing neighboring regions′ POIs for traffic flow prediction, the POI weighting factor *κ* in Eq (9) is alternately set to be 0.9, 0.8, 0.7, 0.6, and 0.5 to check the variation of the performance. The accuracies under different *κ* are shown in Figs 9 and 10 for outflow and inflow prediction, respectively, where the performance curves vary little under different *κ*. This indicates that the neighboring regions′ POIs make little contribution to traffic flow prediction for the target region. This is explained as follows: In practice, people get off only when the taxi arrives at a destination. It happens rarely that people get off in a nearby region and reach a final destination on foot. Nevertheless, the contribution of the neighboring regions′ POIs slightly improves prediction accuracy during different time intervals according to Figs 9 and 10, so we incorporate them into the POI feature in predicting traffic flows.

**Fig 11:**
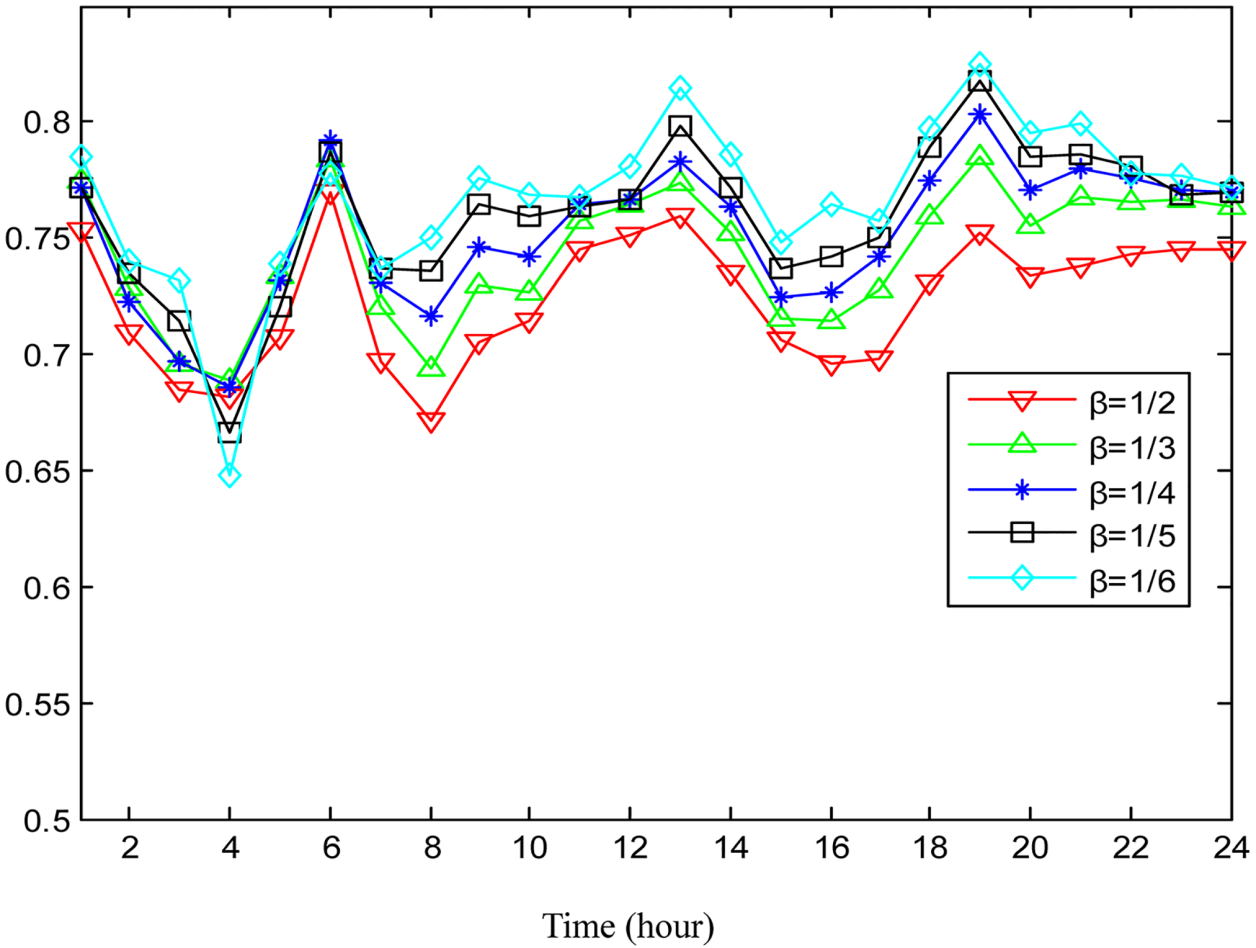
The average prediction accuracies for outflow under different hot degrees determined by *β*.

**Fig 12:**
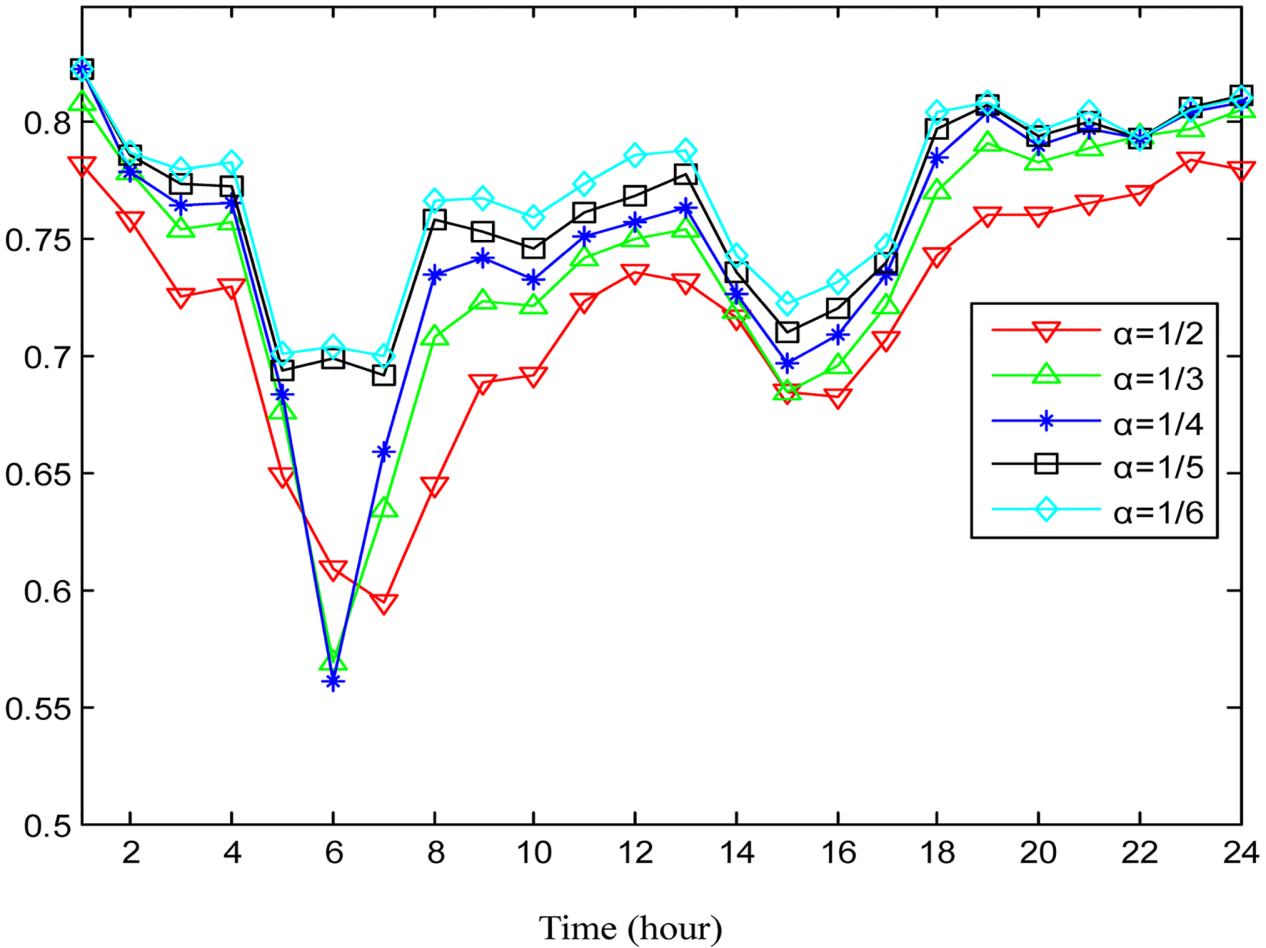
The average prediction accuracies for inflow under different hot degrees determined by *α*.

How the two parameters *α* and *β* for hot region detection affect the performance of traffic flow prediction is also examined. Here, the two parameters are set to be 1/2, 1/3, 1/4, 1/5, and 1/6, alternately. Note that a smaller value of *α* and *β* corresponds with less hot regions but higher hot degrees in terms of traffic flow in accordance with Eqs (3) and (4). As illustrated in Figs 11 and 12, the prediction accuracy improves with the increment of the hot degree for the selected hot regions during the majority of the day.

**Fig 13:**
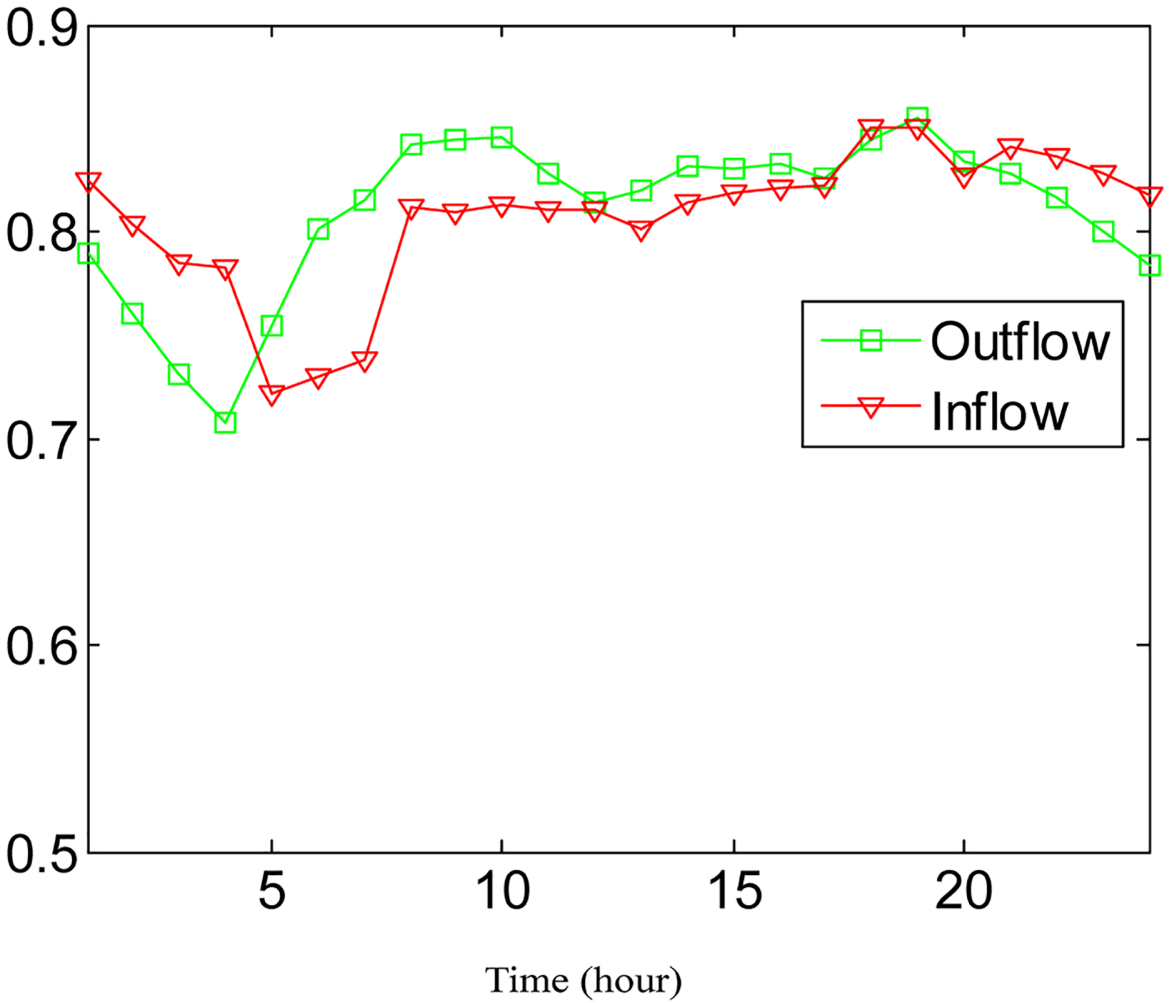
The average prediction accuracies against different time intervals during work days.

**Fig 14:**
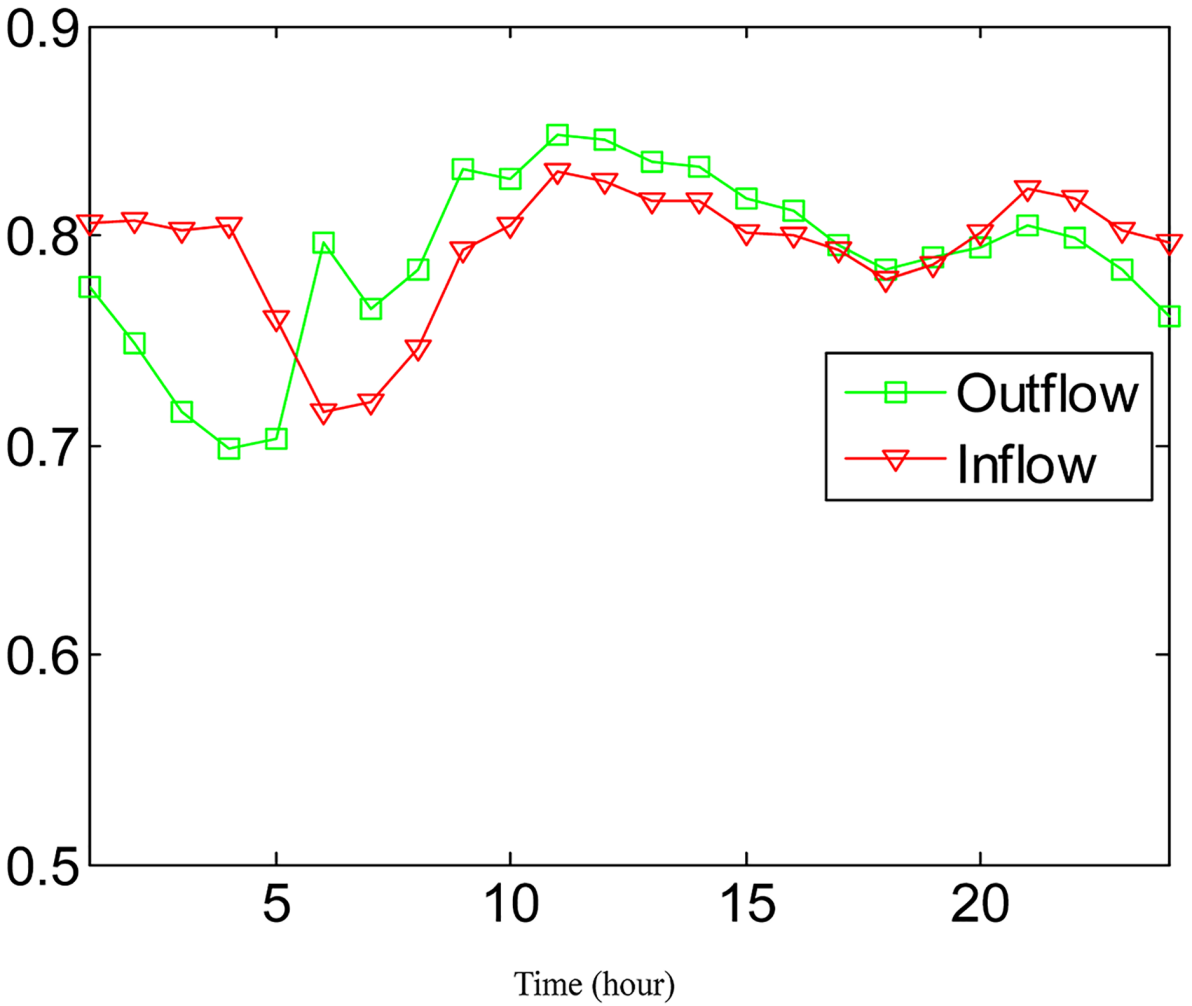
The average prediction accuracies against different time intervals during weekends.

Furthermore, the data for work days and weekends are used separately to evaluate prediction accuracy. First, the data corresponding with work days and weekends are divided into 4 parts at random. Then, the 4−fold Cross Validation method is used for performance evaluation. The average prediction accuracies are shown in Figs 13 and 14. We can see that the fluctuation trend of the prediction accuracy over time in Fig 13 is similar to the case in Fig 14. The overall prediction accuracies for outflow and inflow in different cases are shown in Tables 2 and 3, respectively. From Tables 2 and 3, we can see that the average prediction accuracies for both work days and weekends are better than the case of mixing the two. Therefore, the human mobility patterns for work days and weekends should be different. The overall prediction accuracies for outflow and inflow are 74.49% and 75.15%, respectively.

**Table 2:** The average prediction accuracies (%) on different datasets in the case of outflow.

*α* = *β*	1/2	1/3	1/4	1/5	1/6	Average
**Work days**	77.44	80.24	81.55	82.47	83.17	80.97
**Weekends**	76.65	78.86	79.33	79.55	80.29	78.94
**All days**	71.87	73.93	74.82	75.55	76.28	74.49

**Table 3:** The average prediction accuracies (%) on different datasets in the case of inflow.

*α* = *β*	1/2	1/3	1/4	1/5	1/6	Average
**Work days**	76.75	79.41	81.19	82.61	83.43	80.68
**Weekends**	76.11	78.24	79.35	81.07	82.06	79.37
**All days**	71.43	73.84	75.44	77.08	77.97	75.15

The average prediction accuracies of different week days are shown in Tables 4 and 5 to allow an insight into how general the correlation law holds between human mobility and region functions for each day in a week.

**Table 4:** The average prediction accuracies (%) of outflows for different week days.

*α* = *β*	1/2	1/3	1/4	1/5	1/6	Average
**Mondays**	77.87	81.17	82.00	82.52	83.14	81.34
**Tuesdays**	76.58	79.69	80.36	81.56	81.61	79.96
**Wednesdays**	78.29	82.25	83.24	84.33	84.02	82.43
**Thursdays**	76.49	77.98	81.58	81.77	85.85	80.73
**Fridays**	76.70	80.58	81.45	81.35	81.89	80.40
**Saturdays**	80.24	83.09	84.34	84.67	84.94	83.46
**Sundays**	78.95	81.34	81.63	80.28	80.84	80.61
**Average**	77.87	80.87	82.09	82.35	83.18	81.30

**Table 5:** redThe average prediction accuracies (%) of inflows for different week days.

*α* = *β*	1/2	1/3	1/4	1/5	1/6	Average
**Mondays**	79.31	81.79	83.26	84.02	84.58	82.59
**Tuesdays**	78.54	82.48	83.76	84.29	85.57	82.93
**Wednesdays**	78.95	82.35	84.19	84.34	85.98	83.16
**Thursdays**	77.49	78.75	82.02	81.85	87.09	81.44
**Fridays**	75.81	79.01	79.86	80.15	81.18	79.20
**Saturdays**	80.38	82.37	83.49	83.21	84.54	82.20
**Sundays**	80.71	83.28	83.70	84.36	83.59	83.13
**Average**	78.74	81.43	82.90	83.17	84.65	82.09

As smaller values of *α* and *β* correspond with higher degree of hotness, with the decrement of *α* and *β* in Tables 4 and 5, we can see that the average prediction accuracies of different week days are gradually improved. In Table 4, although the average prediction accuracies of Tuesdays, Thursdays, and Fridays, say, 79.96%, 80.73%, and 80.40%, are a bit smaller than the average prediction accuracy 80.97% of all the work days in Table 2, the average prediction accuracies of Mondays and Wednesdays, namely, 81.34%, 82.43%, are obviously better. In the meantime, the average prediction accuracies of Saturdays and Sundays, 83.46% and 80.61%, are much better than the 78.94% accuracy of the weekends in Table 2. The overall average prediction accuracy in Table 4 is 81.30%, which is almost 7% higher than that of the 74.49% precision in Table 2. In Table 5, only the average prediction accuracy of Fridays, 79.20%, is slightly smaller than the average prediction accuracy of the work days, 80.68%, in Table 3. The average prediction accuracies of the other week days are all 2%-4% higher than that in Table 3. The overall average prediction accuracy in Table 5 is 82.09%, which is almost 7% higher than the 75.15% precision in Table 3.

Note that in this study, we follow the practice of the previous studies in the literature of urban computing [3][4][18] to grant taxi flows as the representative of city-wide human mobility, since the sampling of city mobility by a large number of taxies could approach the trend of peoples′ destinations in a statistical sense under the Law of Large Numbers.

## Comparison

**Fig 15:**
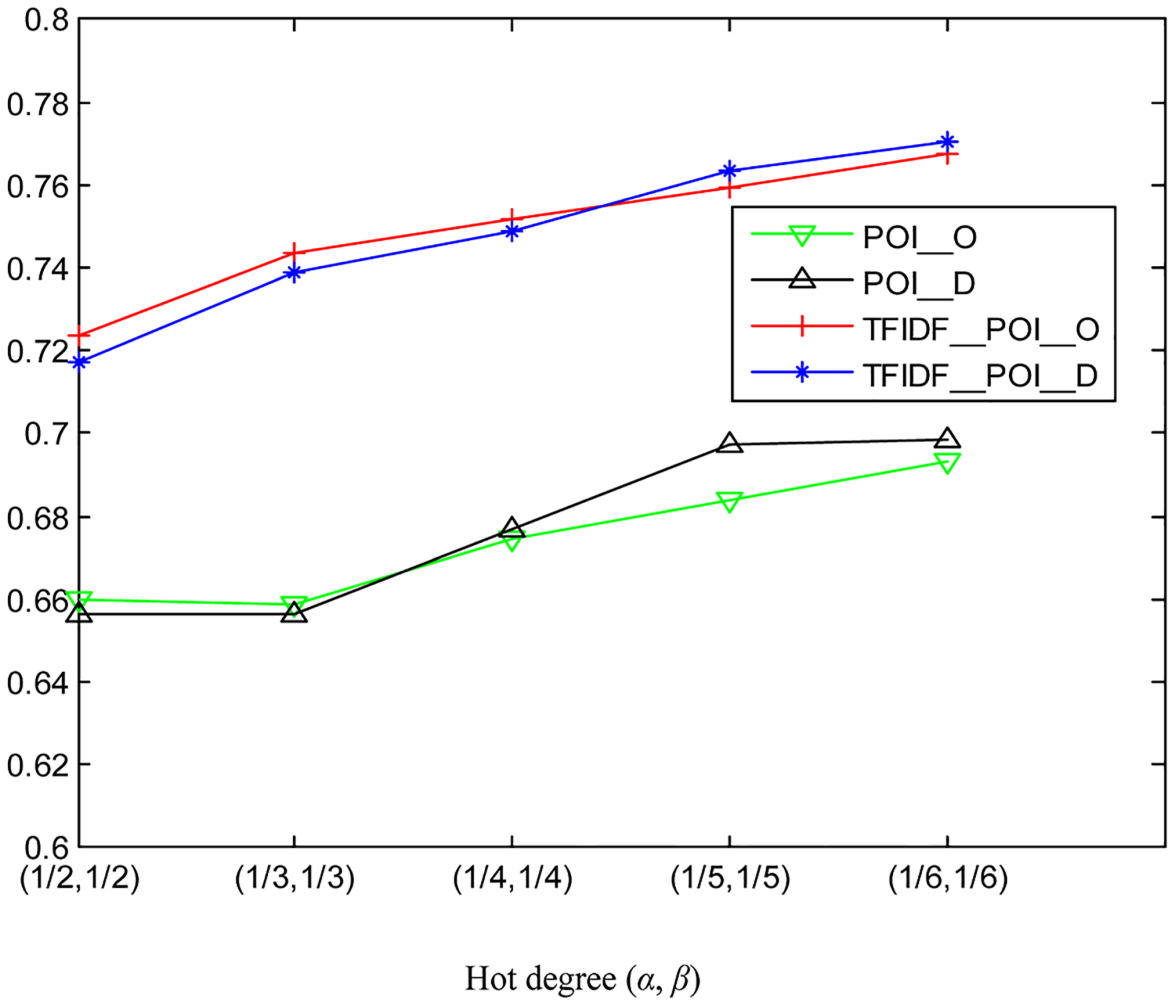
The average prediction accuracies under different hot degrees with and without TF-IDF method.

In the data preprocessing stage, the TF-IDF method is applied to the new region function descriptor. Some experiments have been done in this section to verify the effectiveness of the method. The average prediction accuracies of different time periods under different hot degrees with and without the TF-IDF method are shown in Fig 15. From Fig 15, we can see that all the prediction accuracies with TF-IDF preprocessing are greater than those without TF-IDF preprocessing, which proves the effectiveness of the method.

With historical outflows or inflows as inputs, the averaging method (AM) and the first-order exponential smoothing method (FOESM) are used to estimate $O_{t}^{r}$ and $D_{t}^{r}$ as follows:

$$\begin{array}{c} \begin{array}{c} {\hat{O}}_{t}^{r}=\frac{1}{3}\left(O_{t-1}^{r}+O_{t-2}^{r}+O_{t-3}^{r}\right)\\ {\hat{D}}_{t}^{r}=\frac{1}{3}\left(D_{t-1}^{r}+D_{t-2}^{r}+D_{t-3}^{r}\right) \end{array} \end{array}$$ (15)

$$\begin{array}{c} \begin{array}{c} {\hat{O}}_{t}^{r}=\mu \times O_{t-1}^{r}+\left(1-\mu \right)\times {\hat{O}}_{t-1}^{r}\\ {\hat{D}}_{t}^{r}=\mu \times D_{t-1}^{r}+\left(1-\mu \right)\times {\hat{D}}_{t-1}^{r} \end{array} \end{array}$$ (16)

**Fig 16:**
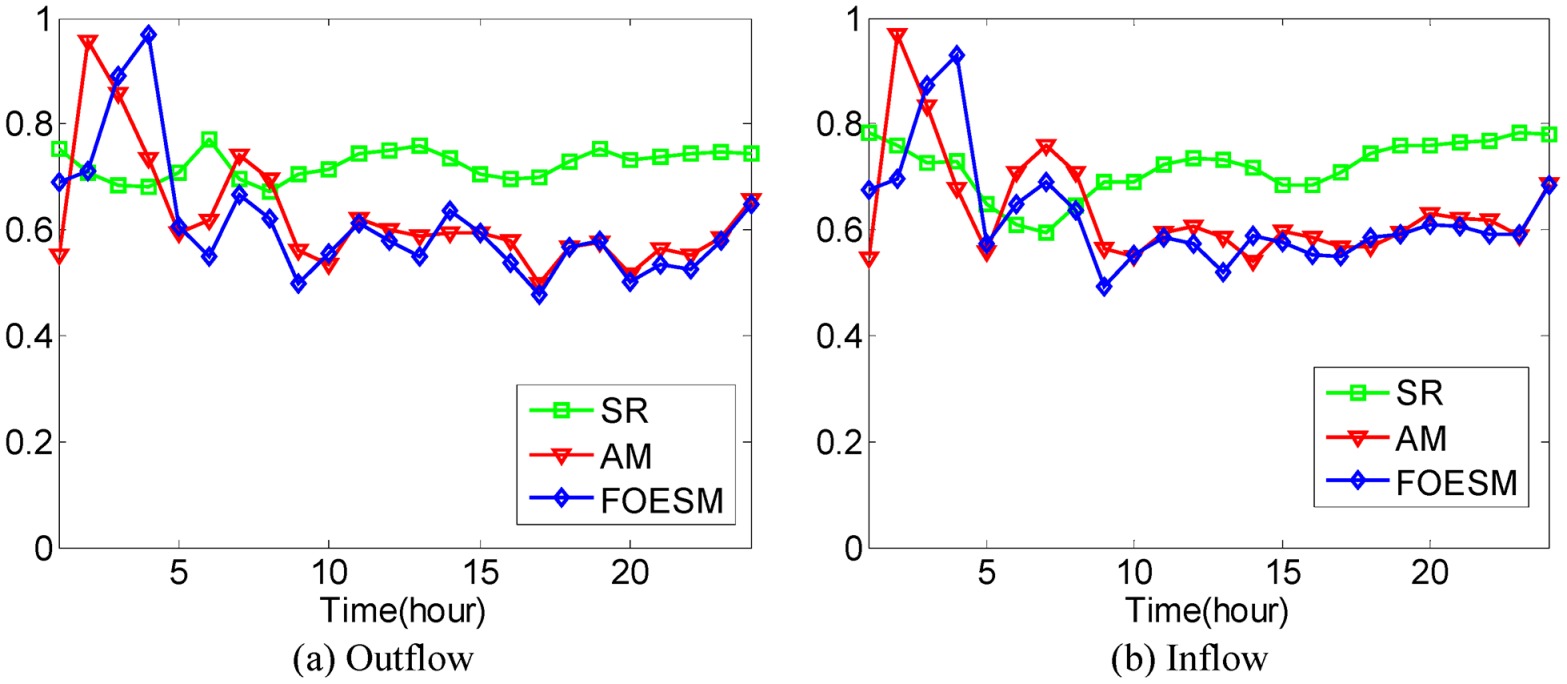
The average prediction accuracies against different time periods under different *κ* with different methods on hot regions with (*α*, *β*)=(1/2, 1/2). (a) The case of outflow; (b) The case of inflow.

**Fig 17:**
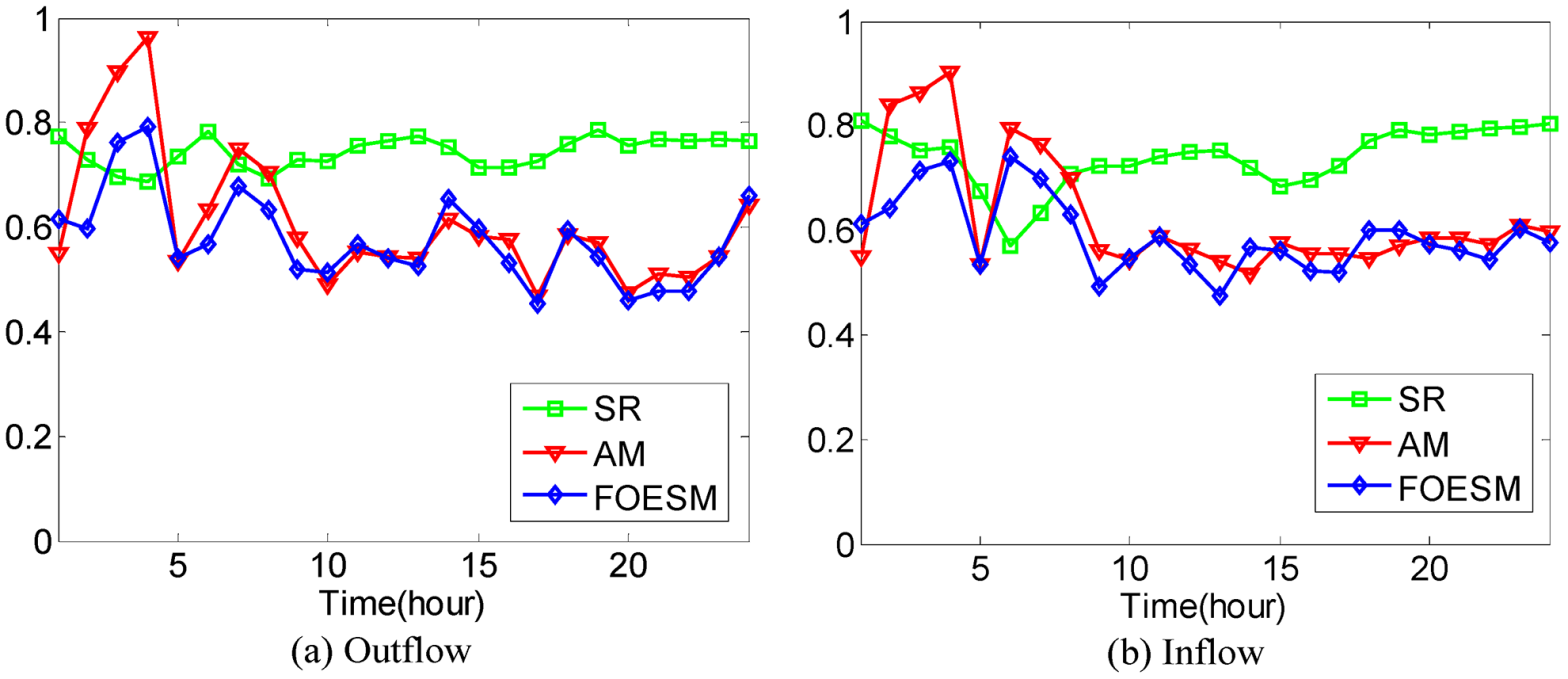
The average prediction accuracies against different time periods under different *κ* with different methods on hot regions with (*α*, *β*)=(1/3, 1/3). (a) The case of outflow; (b) The case of inflow.

**Fig 18:**
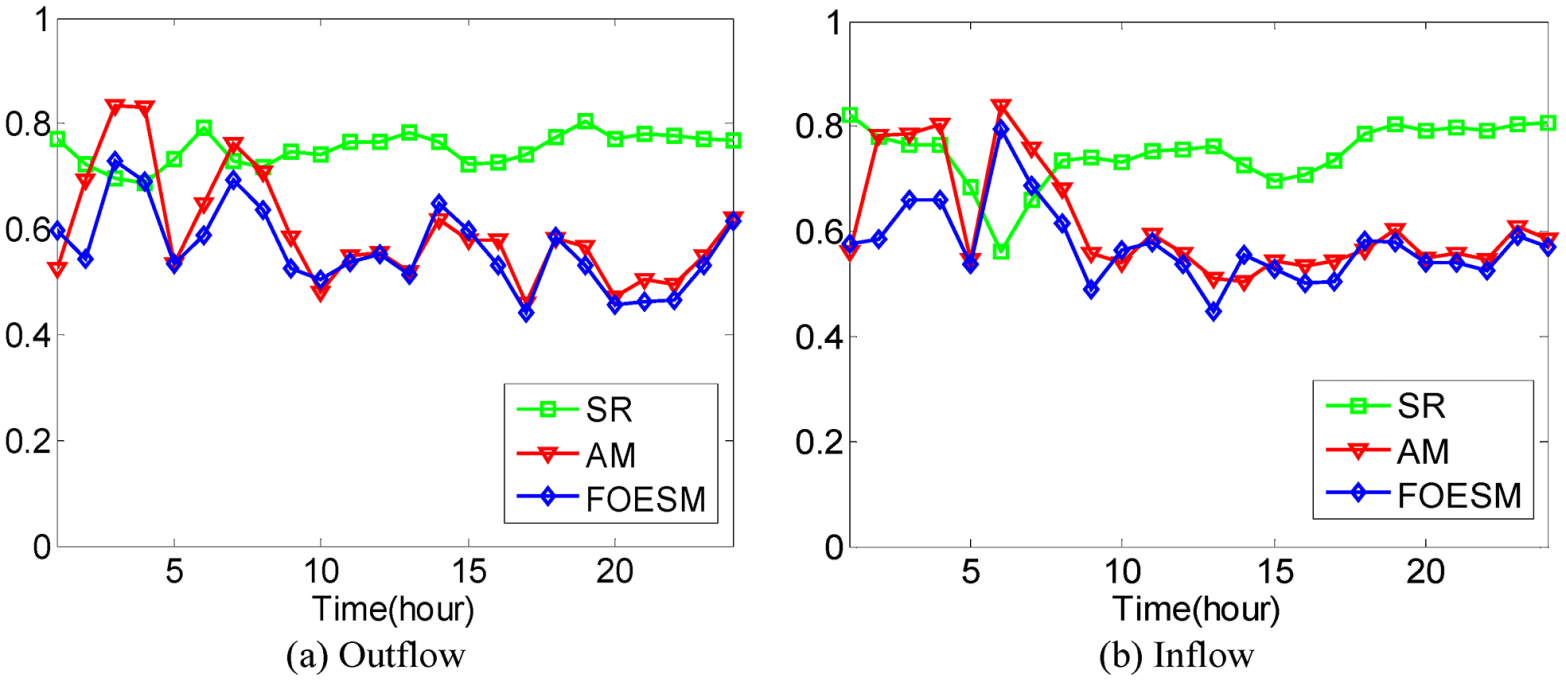
The average prediction accuracies against different time periods under different *κ* with different methods on hot regions with (*α*, *β*)=(1/4, 1/4). (a) The case of outflow; (b) The case of inflow.

**Fig 19:**
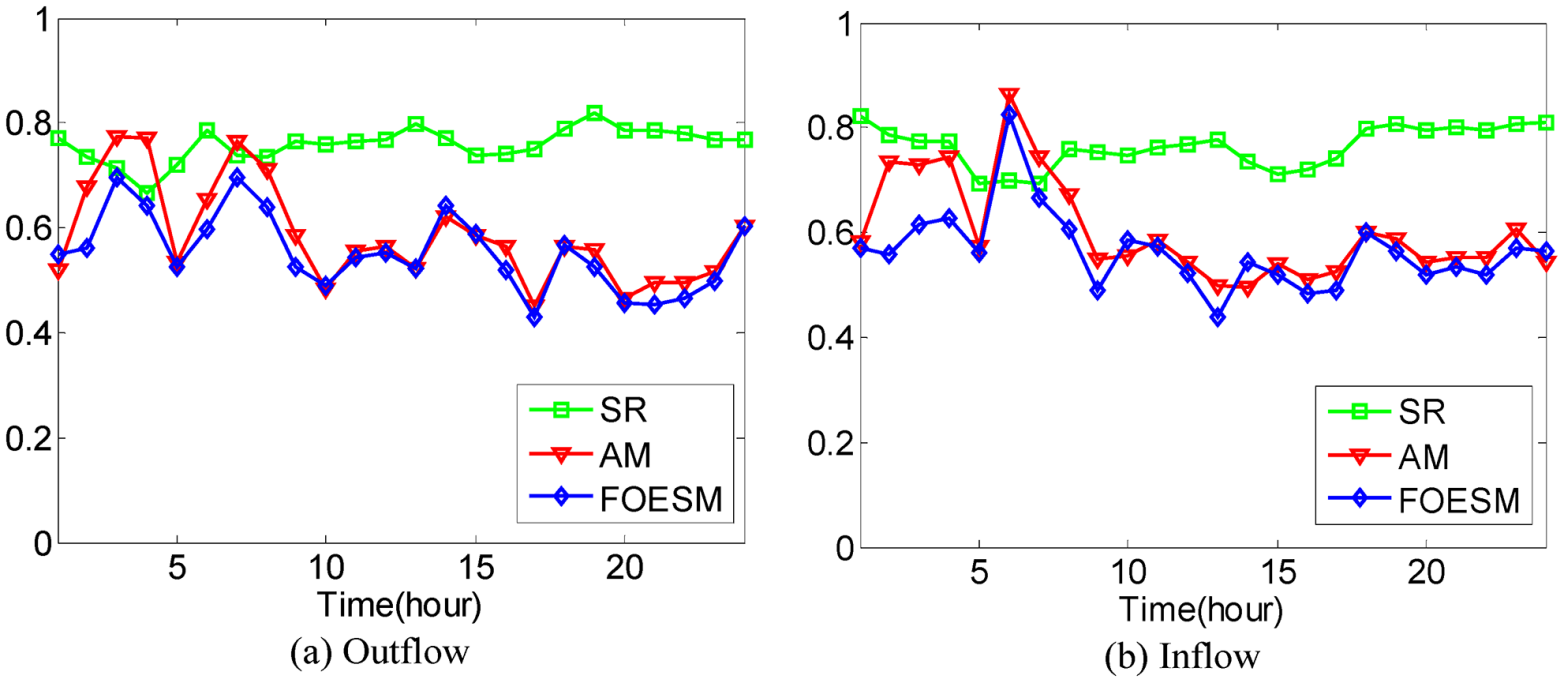
The average prediction accuracies against different time periods under different *κ* with different methods on hot regions with (*α*, *β*)=(1/5, 1/5). (a) The case of outflow; (b) The case of inflow.

**Fig 20:**
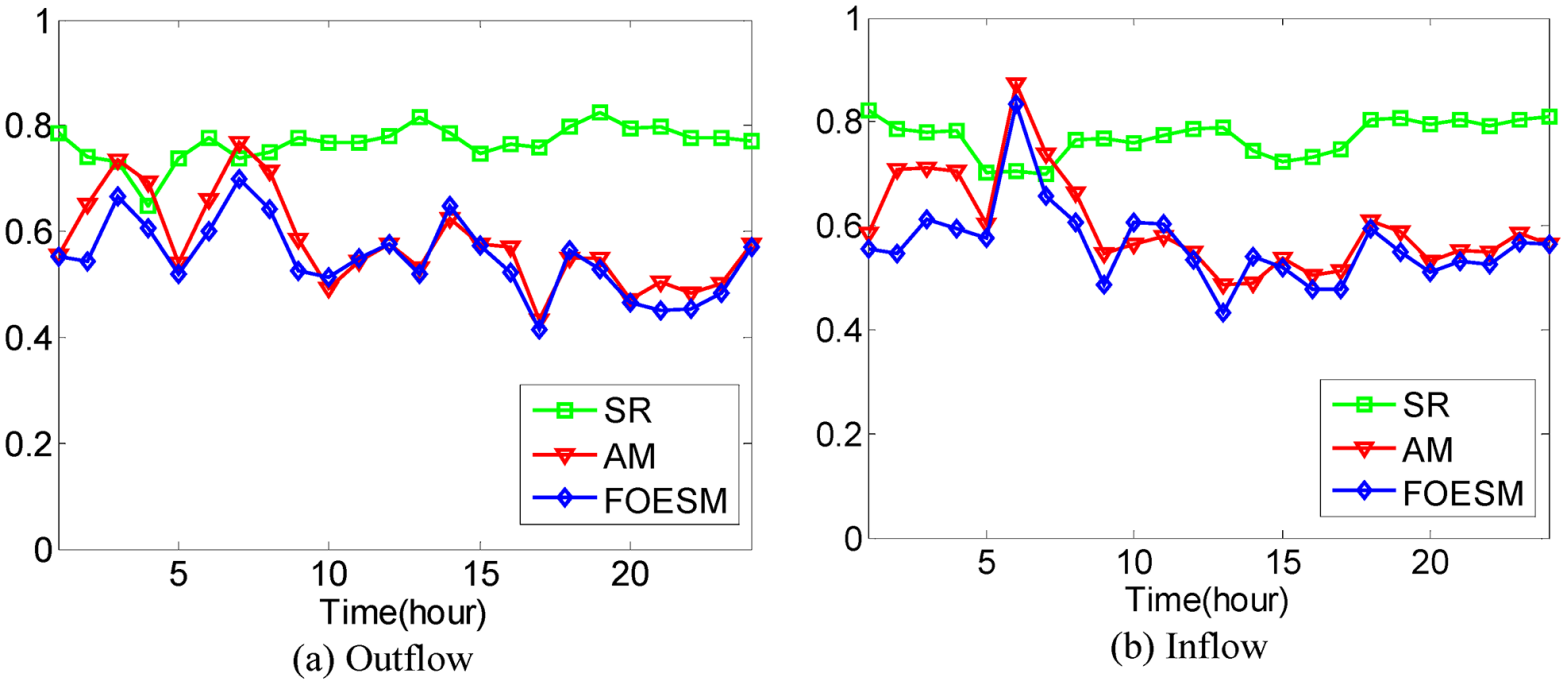
The average prediction accuracies against different time periods under different *κ* with different methods on hot regions with (*α*, *β*)=(1/6, 1/6). (a) The case of outflow; (b) The case of inflow.

In Eq (16), if the value of *μ* is larger, the value of $O_{t-1}^{r}$ and $D_{t-1}^{r}$ has a greater effect on the predicted value. We set ${\hat{O}}_{0}^{r}=O_{0}^{r}$ and ${\hat{D}}_{0}^{r}=D_{0}^{r}$ during the experiments. After that, the Mean Absolute Percentage Error method as shown in Eqs (13) and (14) is used to evaluate the prediction accuracy with 4-fold Cross Validation. In the experiments, it is found that when the value of *μ* is set to be 0.5, 0.6, 0.5, 0.5, 0.9, the best average prediction accuracy can be approached under different *κ* on the datasets of hot regions with (*α*, *β*)=(1/2, 1/2), (1/3, 1/3), (1/4, 1/4), (1/5, 1/5), (1/6, 1/6). The best average prediction accuracies of inflow and outflow with different methods are shown in Figs 16–20.

From Figs 16–20, we can see that the average prediction accuracies of the proposed method are mostly higher than those of the other two methods. The only exception is the time period from 1:00 AM to 7:00 AM, as few people travel during that time, which makes the OD data ill-posed. After that time, increasingly many people travel between urban regions, and the OD data return to the normal case. For AM and FOESM, the average prediction accuracies of different time periods exhibit a decreasing trend and fluctuate around 0.5. In contrast, the average prediction accuracies of the proposed method are around 0.7 in different time periods, which is remarkably higher than that of AM and FOESM while exhibiting less fluctuation. We know that smaller values of *α* and *β* correspond with higher degree of hotness of hot regions. As shown in Figs 16–20, during the time period from 1:00 AM to 7:00 AM, with the decrease of *α* and *β*, the prediction accuracies of AM and FOESM gain fewer advantages over the proposed method.

The results shown in Figs 16–20 confirm that the proposed method promises a better overall performance in terms of both prediction accuracy and stability compared with AM and FOESM.

**Fig 21:**
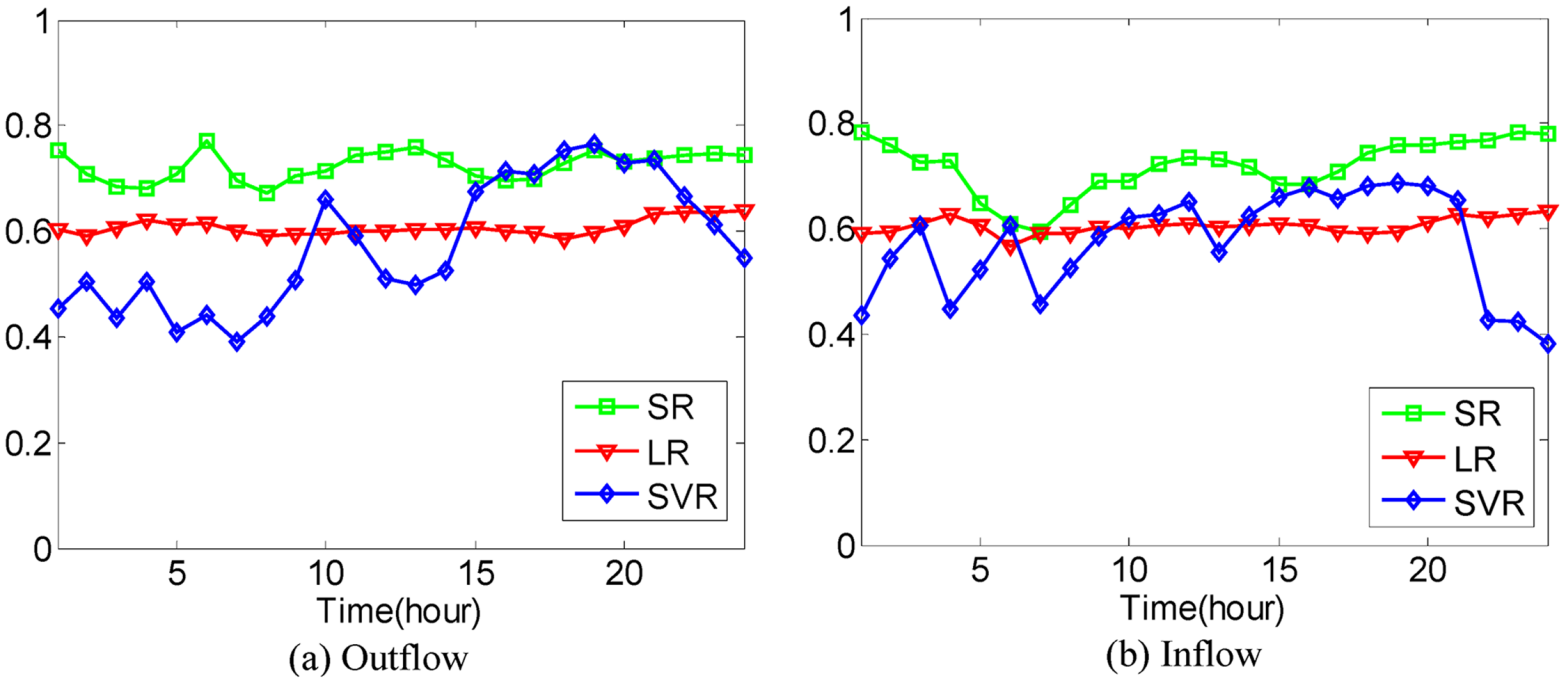
The average prediction accuracies against different time periods under different *κ* with different models on hot regions with (*α*, *β*)=(1/2, 1/2). (a) The case of outflow; (b) The case of inflow.

**Fig 22:**
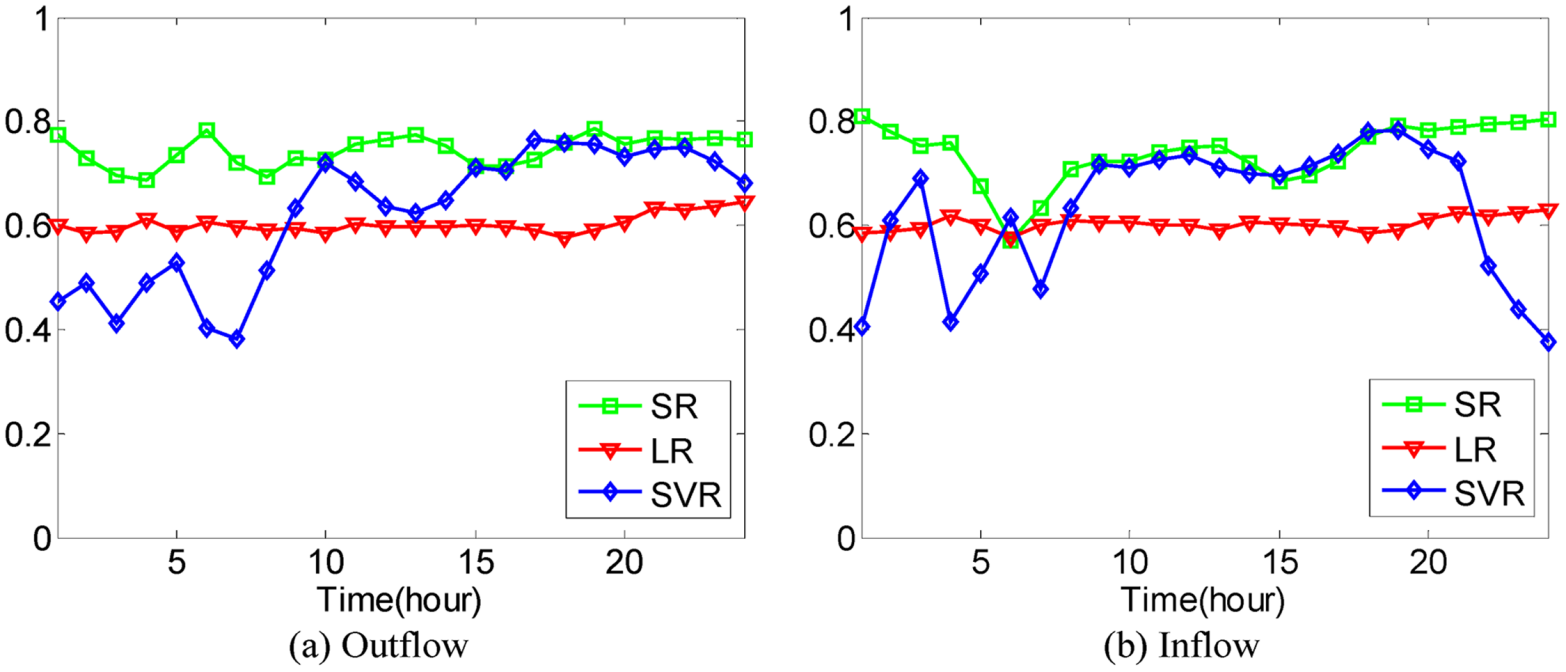
The average prediction accuracies against different time periods under different *κ* with different models on hot regions with (*α*, *β*)=(1/3, 1/3). (a) The case of outflow; (b) The case of inflow.

**Fig 23:**
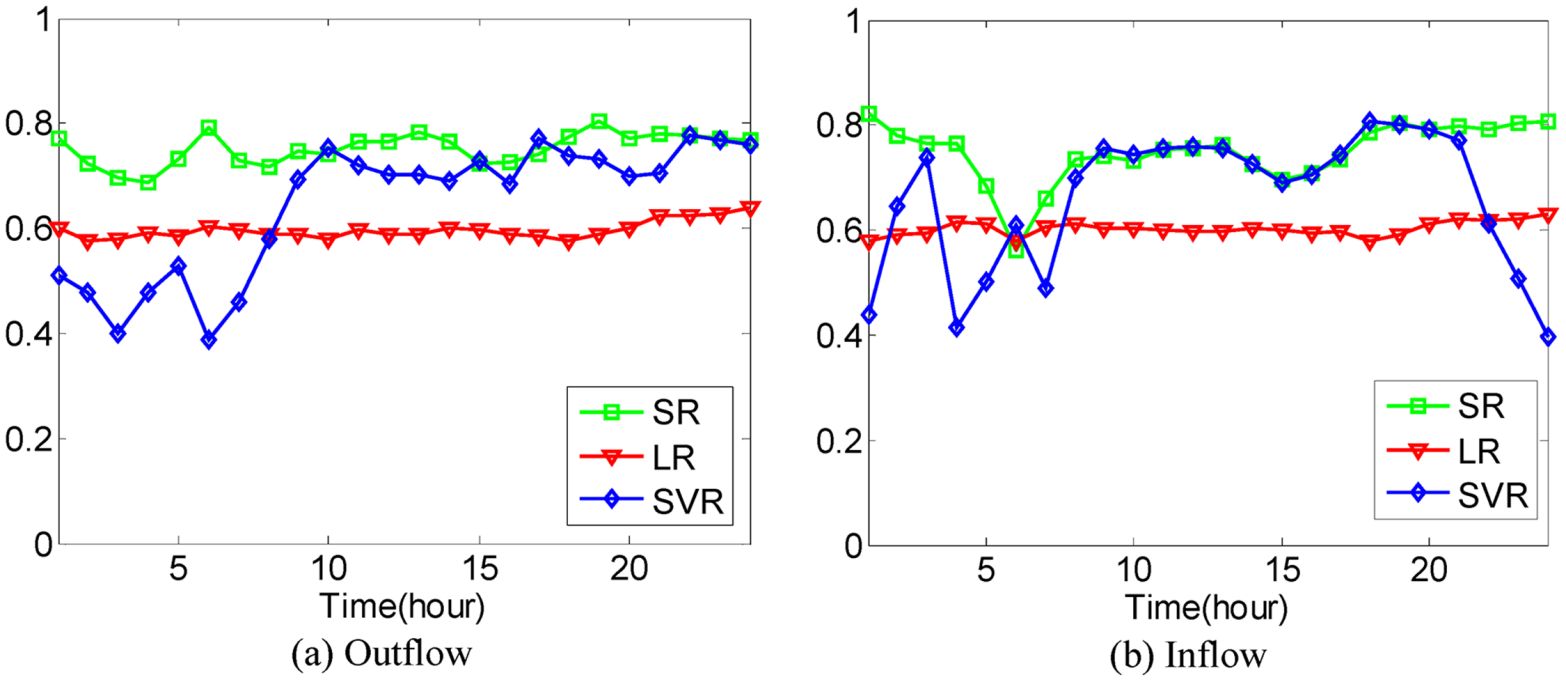
The average prediction accuracies against different time periods under different *κ* with different models on hot regions with (*α*, *β*)=(1/4, 1/4). (a) The case of outflow; (b) The case of inflow.

**Fig 24:**
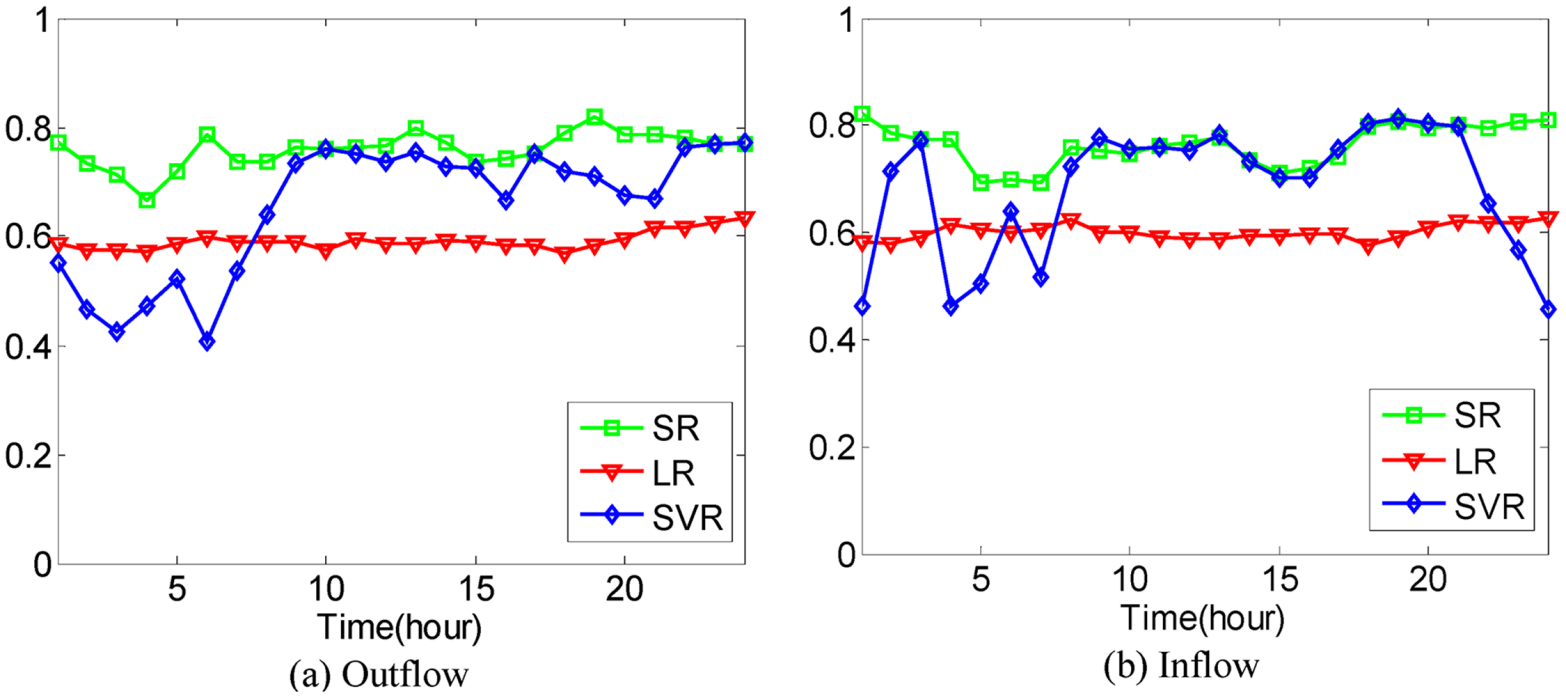
The average prediction accuracies against different time periods under different *κ* with different models on hot regions with (*α*, *β*)=(1/5, 1/5). (a) The case of outflow; (b) The case of inflow.

**Fig 25:**
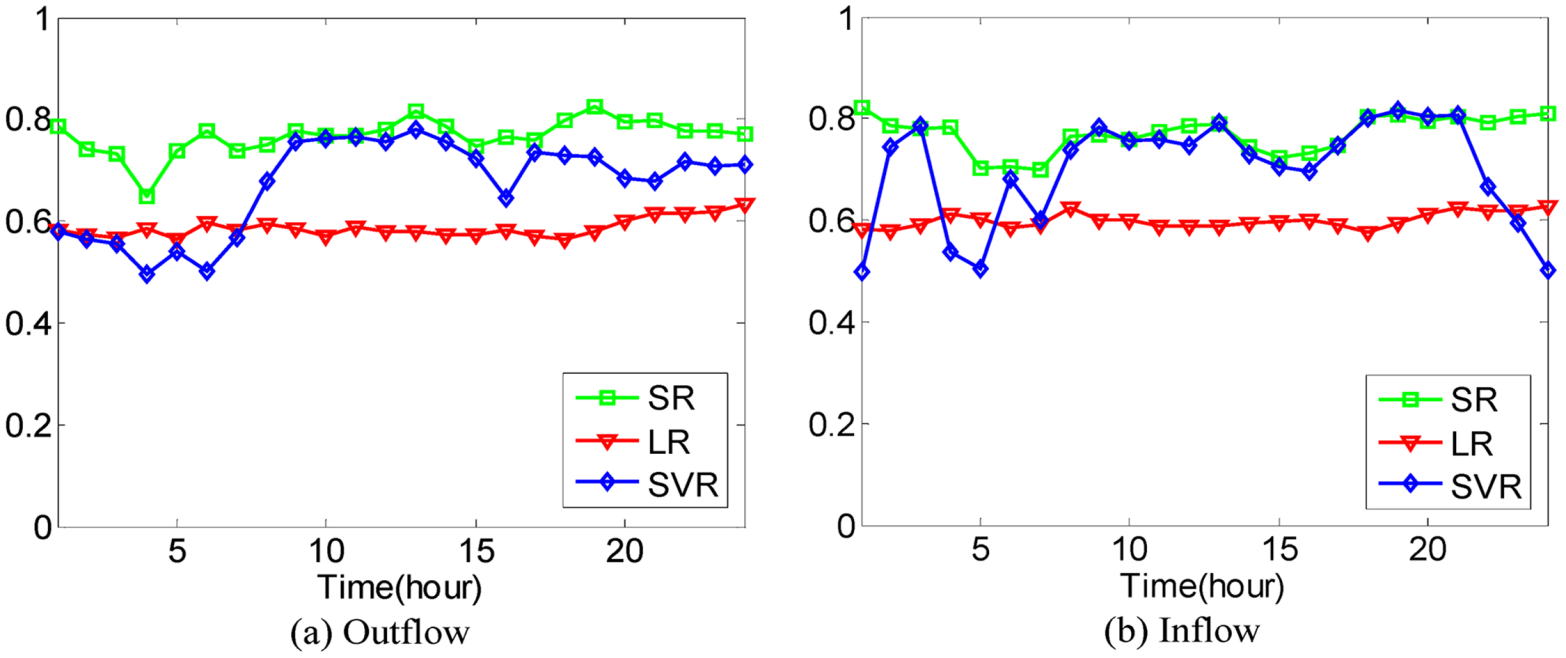
The average prediction accuracies against different time periods under different *κ* with different models on hot regions with (*α*, *β*)=(1/6, 1/6). (a) The case of outflow; (b) The case of inflow.

In addition to the above comparison between different methods, taking all 20−dimensional POIs as inputs, Linear Regression (LR) model and SVR (Support Vector Regression) model are used to predict the traffic flows of hot regions for the sake of comparison with Sparse Representation (SR), where the polynomial kernel is applied in SVR. Performance evaluation is conducted in the sense of Eqs (13) and (14) with the 4-fold Cross Validation. The average prediction accuracies using different models under different cases are shown in Figs 21–25.

From Figs 21–25, we can see that although the LR model has the best stability and prediction accuracies under different cases, around 0.6, its prediction accuracies under different cases are much smaller than those of the proposed SR model, which has prediction accuracies under different cases of around 0.7 in different time periods. The prediction accuracies of the SVR model are much higher than those of the LR model in most time periods but still lower than those of the proposed SR model. The proposed SR model also has better stability than the SVR model under different cases.

In this study, the sparse representation method is used to select the POIs that are relevant to the traffic flows of hot regions, acting as feature selection. It is generally acknowledged that SVR is a much stronger prediction algorithm compared to LR model but its performance is not so good here. We attribute this to the lack of variable selection. Therefore, SR model and SVR model are combined to predict traffic flows. First, SR model is used to select the POIs relevant to the traffic flows of hot regions. Then, the selected POIs are taken as inputs and SVR model is used to predict the traffic flows of hot regions. Following 4-fold cross validation, the average prediction accuracies of different methods are shown in Tables 6 and 7.

**Table 6:** The average prediction accuracies (%) for outflow prediction using different methods.

*α* = *β*	1/2	1/3	1/4	1/5	1/6	Average
**SVR**	61.58	66.32	69.25	69.63	71.56	67.67
**SR**	71.87	73.93	74.82	75.55	76.28	74.49
**SR+SVR**	76.23	76.68	76.99	76.83	76.25	76.60

**Table 7:** The average prediction accuracies (%) for inflow prediction using different methods.

*α* = *β*	1/2	1/3	1/4	1/5	1/6	Average
**SVR**	61.70	69.10	71.86	73.72	74.15	70.11
**SR**	71.43	73.84	75.44	77.08	77.97	75.15
**SR+SVR**	74.78	77.75	78.23	78.04	78.08	77.38

From Tables 6 and 7, we can see that the average prediction accuracies of the combined SR and SVR model in the cases of outflow and inflow are 76.60% and 77.38%, respectively, which are almost 10%-17% higher than that of SVR model, and 2% higher than SR model. The above results show that variable selection as promised by the SR model is critical for understanding the truly contributive POIs in attracting traffic flows.

## Conclusion

This study aims to find how urban planning affects human mobility. The discovered relation between city planning and human mobility is important for transportation and business intelligence, which can act as guidelines to design rather than manage traffic at the urban planning stage while boom commercial activeness through region function design from an ecosystem point of view.

In this paper, we proposed a method that can predict the population outflows and inflows for a region only with the numbers and categories of POIs. The contributions of this study lie in the following aspects: (1) As we see, the best way to control traffic jams is to design traffic flows at the urban planning stage since when city infrastructures and travel behaviors are fixed, we are limited in what we can do to alleviate traffic jams. Therefore, human mobility prediction from city functions is a novel and significant problem has not attracted attention so far. This paper aims to study the possibility of predicting traffic flows entering and leaving a region based on the city functions supplied by this region. To the best of our knowledge, this should be the first endeavor to study predictive urban planning in terms of optimizing traffic flows. In the meantime, human mobility is a major issue to be taken into account when planning commercial areas. This study can provide predictive view regarding how to attract people′s visiting by designing region functions. (2) Unlike existing studies, this work aims to find out the motivations that cause human mobility as well as the impact factor of every causal. Here, we propose applying sparse representation to a linear predictor to find the contribution of every category of city function in causing people to visit a place. By solving the predictor in the sense of sparse representation, we find that average prediction precision is over 74% and each type of POI contributes differently in the predictor, which accounts for the different reasons as well as degrees for attracting people. Moreover, the discovered factors that have high impacts on people′s mobility are valuable in that they can act as the input variables to a variety of predictors to make prediction of human mobility from region functions possible. As confirmed by the experiments, SVR model performs poorly without variable selection to identify the relevant factors affecting human mobility. In contrast, its performance can be greatly improved by applying the selected variables resulting from sparse representation.

The main lessons about human mobility learnt from this study are as follows: (1) The factors highly contributive to human mobility vary from time to time. During the morning rush hour, outflows are mainly from residential areas, restaurants, and hotels, while the destinations are mostly corporations, financial service agencies, and some well-known addresses. For lunch time and dinner time, notably, the main origin is corporation/business, and the main destination is restaurants. At night, the main traffic flows are from restaurants to residential areas and hotels. (2) Urban human mobility patterns in a week day could be different compared to the rest of the week days. The aforementioned findings should be meaningful in understanding urban human mobility patterns for urban planning, where the contribution of each type of city function in terms of attracting people to a region can be quantitatively identified such that the overall effect of region planning can be predicted.

This research is at the very beginning. There should be a lot of future works to be done to improve the work by considering additional factors, such as weather, traffic control, and social event. Besides, more nonlinear predictors should be evaluated in comparison with the proposed method. Furthermore, more data of different cities should be applied to validate the proposed method. Taking into account climate, urban traffic should not be the same in different months. In the follow-up study, we will collect more data for different months to validate the proposed model.
